# Highly Emissive Far Red/Near‐IR Fluorophores Based on Borylated Fluorene–Benzothiadiazole Donor–Acceptor Materials

**DOI:** 10.1002/chem.201602010

**Published:** 2016-07-27

**Authors:** Daniel L. Crossley, Inigo Vitorica‐Yrezabal, Martin J. Humphries, Michael L. Turner, Michael J. Ingleson

**Affiliations:** ^1^ School of Chemistry University of Manchester Manchester M13 9PL UK; ^2^ Cambridge Display Technology Ltd. (Company Number 02672530) Unit 12 Cardinal Park, Cardinal Way Godmanchester PE29 2XG UK

**Keywords:** boron, chromophores, cross-coupling, donor–acceptor systems, near-infrared fluorescence

## Abstract

Stille, Suzuki–Miyaura and Negishi cross‐coupling reactions of bromine‐functionalised borylated precursors enable the facile, high yielding, synthesis of borylated donor–acceptor materials that contain electron‐rich aromatic units and/or extended effective conjugation lengths. These materials have large Stokes shifts, low LUMO energies, small band‐gaps and significant fluorescence emission >700 nm in solution and when dispersed in a host polymer.

## Introduction

Near‐infrared (NIR) absorbing and emissive molecules have attracted significant interest due to potential applications including night‐vison displays, sensors and in‐vivo imaging.^1^, ^2^ Generating organic NIR emitters that have appreciable quantum yields is challenging due to the energy gap law where the emission efficiency reduces with decreasing energy gap.^3^ This is exacerbated in the solid state as aggregation often leads to additional non‐radiative pathways and significant emission quenching.^4^ One effective strategy for accessing low‐band‐gap materials is to construct π‐conjugated systems containing donor (D) and acceptor (A) groups.^5^ However, d–A materials with appreciable solid state NIR emission are currently rare,^2^ therefore new low‐band‐gap d–A materials are desirable, particularly if accessible by simple modular routes.

Another attractive property of D–A materials is the large degree of control over the electronic and optical properties by rational structural modification. For example, the coordination of a Lewis acid to a Lewis basic site on an acceptor moiety has been previously used to generate D–A materials with low LUMO energies and small band‐gaps.^6^ A related approach that generates D–A materials that are more stable to moisture and Lewis bases appends BR_2_ moieties to a conjugated framework through formation of a C−B bond and a dative bond from the acceptor to the boron centre.^7^ We have recently utilised this methodology to synthesise borylated 9,9‐dioctylfluorene‐benzothiadiazole (F8‐BT) containing d–A materials by directed electrophilic borylation which concomitantly forms a C–B and a N→B bond.^8^ This borylation reaction planarises the conjugated backbone generating rigid structures with extended effective conjugation lengths. These fused materials showed large Stokes shifts, low band‐gaps, were stable to H_2_O and O_2_ and were highly emissive in solution and the solid state; for example, F8‐BT‐F8 functionalised with a BPh_2_ group was highly emissive in the solid state (PLQY 33 % with *λ*_max_=696 nm).^8^ Whilst notable it is desirable to develop synthetic routes to rapidly access analogues with lower band‐gaps that were highly emissive in the solid state with emission further into the NIR (*λ*_max_≫700 nm).

**Scheme 1:**
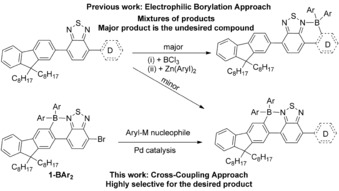
The formation of borylated F8‐BT units with appended donor units by electrophilic borylation (previous work) and by cross‐coupling (this work).

A simple approach to deliver these materials is to introduce more electron‐rich donor units than F8 and/or to extend the effective conjugation length. However, coupling other donor units to F8‐BT generates unsymmetric F8‐BT‐D materials that complicates the electrophilic borylation step, particularly where electron‐rich aromatic units such as thiophene are employed as these groups undergo C−H borylation preferentially to the F8 moiety (Scheme 1, top).^8^, ^9^ This is undesirable as thiophene‐BT compounds borylated on thiophene are significantly less emissive than the borylated F8‐BT analogues.^8^ An alternative method to generate compounds containing both electron‐rich aromatic and borylated F8‐BT units (Scheme 1, bottom) is by Pd catalysed cross‐coupling post borylation. Including a suitable functional group is therefore essential for the synthesis of these materials through directed electrophilic borylation (producing **1‐BAr_2_** Scheme 1, bottom left) and subsequent cross‐coupling. Whilst there is significant precedence for a range of boron‐containing compounds participating in Stille couplings with retention of the boron moiety,^7^, ^10^ precedence for the more desirable (from a toxicity perspective) Suzuki–Miyaura cross‐couplings that proceed with retention of a four coordinate boron moiety is much more limited.7c This is presumably due to the reaction conditions in the latter (particularly the necessity for aqueous base) leading to deborylation.1

Herein we report the development of methods to form D‐BT‐F8 and D‐(BT‐F8)_2_ materials selectively borylated on the F8 unit and show that they are strongly emissive in solution and when dispersed in a host polymer, with *λ*_max_>700 nm and display good quantum yields for emission in the NIR region.

## Results and Discussion

**Figure 1:**
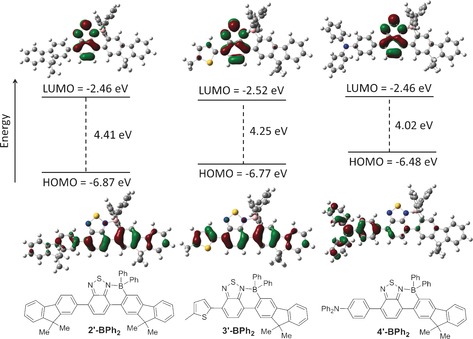
Molecular orbital energy levels and molecular orbital contours (isovalue=0.04) of the HOMO and LUMO of **2′‐BPh_2_** (left), **3′‐BPh_2_** (middle) and **4′‐BPh_2_** (right).

To explore the hypothesis that appending more electron‐rich donor units onto **1‐BPh_2_** has the desired outcome of lowering the band‐gap calculations were performed on three model compounds (octyl groups replaced with Me in each case, Figure 1) **2′‐BPh_2_**, **3′‐BPh_2_**, and **4′‐BPh_2_**, at the M06‐2X/6311G(d,p) level with PCM solvation (DCM). In each case these calculations indicated that the LUMO is predominantly localised on the BT moiety whilst the HOMO is more delocalised. The HOMO in **3′‐BPh_2_** is the most delocalised of the series having more character on the non‐borylated aromatic relative to **2′‐BPh_2_** and more character on the borylated BT‐F8 moiety relative to **4′‐BPh**_2_. The greater HOMO delocalisation in **3′‐BPh_2_** is consistent with this compound having a more planar backbone (dihedral angles between the thiophene and BT rings in **3′‐BPh_2_** are ca. 20 ° whereas for **2′‐BPh_2_** and **4′‐BPh_2_** analogous dihedral angles are between 34–40 °). A greater localisation of the HOMO on the Ph_2_N‐C_6_H_4_ moiety in **4′‐BPh_2_** is apparent and consistent with the more electron‐rich nature of this triphenylamine relative to the fluorene. Relative to the HOMO energy of **2′‐BPh_2_** the HOMO for **3′‐BPh_2_** is 0.12 eV higher and that for **4′‐BPh_2_** 0.39 eV higher in energy, indicating that a red‐shift in absorption and emission is indeed feasible by appending electron‐rich aromatics onto **1‐BPh_2_**. It is notable that whilst there is a significant change in the HOMO energy level for **4′‐BPh_2_** the LUMO energy level is identical to that calculated for **2′‐BPh_2_**. The minor reduction in the LUMO energy for **3′‐BPh_2_** is also consistent with greater delocalisation enabled by the more planar structure.1

**Scheme 2:**
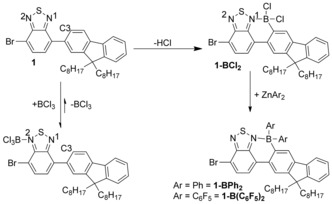
The formation of **1‐BAr_2_** by electrophilic borylation.

With the calculations confirming the validity of our approach, brominated precursor **1** was borylated at C3 to produce **1‐BCl_2_** (not isolated) by using an excess of BCl_3_ (ca. 5 equiv) and 16 h reaction time. The extended reaction time (relative to the rapid borylation of F8‐BT‐F8)^8^ is necessary due to preferential coordination of BCl_3_ to N2 (Scheme 2), that significantly reduces the nucleophilicity of the remaining nitrogen N1 preventing coordinating of BCl_3_ to this nitrogen that leads to directed C−H borylation. Binding of BCl_3_ to N2 appears to be reversible as the C−H electrophilic borylation does proceed with evolution of HCl that can be removed from solution under a dynamic flow of nitrogen (volatile BCl_3_ is removed from the reaction mixture along with HCl gas under the dynamic flow of nitrogen thus an excess of BCl_3_ is required to maintain sufficient solution concentration over 16 h). Compound **1‐BCl_2_** can be functionalised in‐situ with ZnPh_2_ or Zn(C_6_F_5_)_2_, using the previously reported methodologies,7c, 8 to produce **1‐BPh_2_** and **1‐B(C_6_F_5_)_2_**, in good isolated yields (72 and 96 %, respectively).2

**Figure 2:**
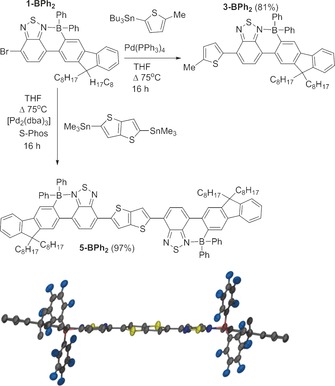
Stille cross‐coupling conversions of **1‐BPh_2_** to **3‐BPh_2_** and **5‐BPh_2_**. Bottom: solid state structure of **5‐B(C_6_F_5_)_2_** (hydrogen atoms are omitted for clarity and only the first carbon of the octyl substituents shown), thermal ellipsoids at the 50 % probability level.

Compounds **1‐BAr_2_** were stable to the Stille cross‐coupling reaction conditions utilised herein with cross‐coupling of **1‐BPh_2_** with 2‐methyl‐5‐(tri‐*n*‐butylstannane)thiophene resulting in the formation of **3‐BPh_2_** in good yield (81 %, Figure 2). This gives an unsymmetrically substituted D_1_–BT–D_2_ system that is exclusively borylated on the less nucleophilic aromatic unit (F8), selectivity not achievable through directed electrophilic borylation of thiophene‐BT‐F8.^8^ This strategy can be extended by the reaction of **1‐BPh_2_** with a bis‐stannane, such as 2,5‐bis(trimethylstannyl)thieno[3,2‐*b*]thiophene. This coupling links two borylated F8‐BT units by a sterically unencumbered π‐conjugated spacer (thieno[3,2‐*b*]thiophene; TT) to give **5‐BPh_2_** (Figure 2). The incorporation of a sterically unhindered π‐conjugated spacer unit was selected to maintain good planarity between the two conjugated borylated aromatic frameworks thereby extending the effective conjugation length.^11^, 3

**Scheme 3:**
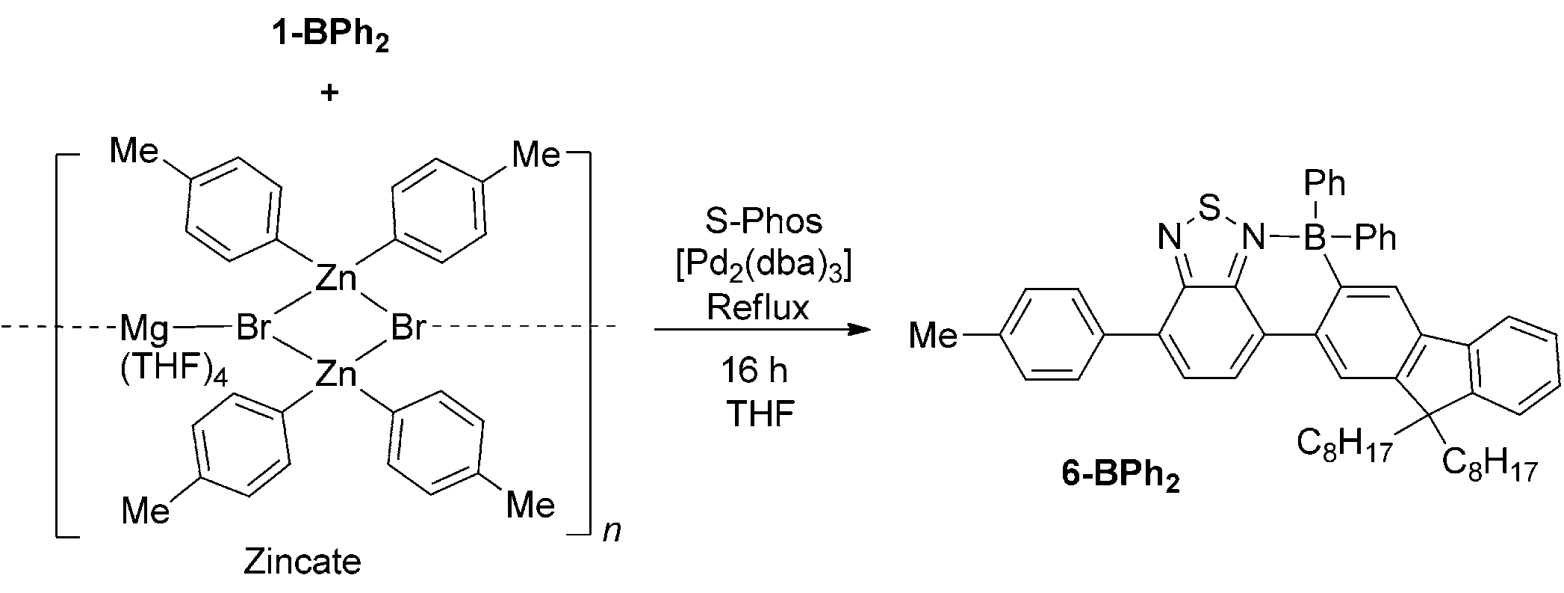
Negishi cross‐coupling of **1‐BPh_2_**.

Whilst **5‐BPh_2_** did not prove amenable to crystallisation, crystals of its bispentafluorophenyl‐substituted congener **5‐B(C_6_F_5_)_2_**, were accessible (made through an analogous Stille coupling approach albeit in lower yield).^12^ The solid state structure of **5‐B(C_6_F_5_)_2_** (Figure 2) demonstrated high co‐planarity between the F8‐BT borylated units and the bridging thieno[3,2‐*b*]thiophene (angle between the BT and thieno[3,2‐*b*]‐thiophene plane is 8.7 °). Bond metrics for the boracycle are unremarkable and comparable to the previously reported borylatively fused structures.^8^ Compounds **5‐BAr_2_** contain four quaternary centres and examination of the extended packing structure of **5‐B(C_6_F_5_)_2_** reveal the absence of close π–π stacking contacts between the planar conjugated cores of adjacent molecules confirming the importance of the quaternary centres in preventing aggregation in the solid state.2

Whilst cross‐coupling reactions using stannanes are prominent in the synthesis of thienyl d–A systems due to their reliability, drawbacks exist particularly associated with the toxicity of organostannane compounds. Negishi cross‐coupling reactions utilise nontoxic and highly nucleophilic organozinc reagents.^13^ The application of Negishi cross‐coupling to **1‐BPh_2_** using a previously reported zincate as the transmetalation reagent^14^ results in the formation of **6‐BPh_2_** in an excellent isolated yield (98 %) with the workup a simple filtration through silica gel (Scheme 3). This material is not studied further herein as its band‐gap will be larger than that of **2‐BPh_2_** based on *p*‐tolyl being a weaker donor moiety than F8, but it does confirm that Negishi protocols are compatible with the borylated moiety.

**Scheme 4:**
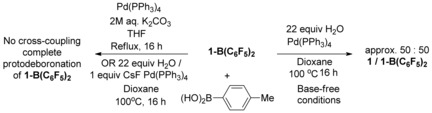
Protodeboronation of **1‐B(C_6_F_5_)_2_** with and without base.

Suzuki–Miyaura cross‐coupling is generally preferable to both Stille and Negishi cross‐coupling due to its advantageous features which include air‐stability of the transmetalation reagent, mild aqueous reaction conditions, high functional group tolerance and low toxicity of reaction by‐products.^15^ In initial screening reactions the synthesis of **6‐BAr_2_** from **1‐BAr_2_** by conventional Suzuki–Miyaura cross‐coupling reaction conditions (e.g., boronic acid/etherate solvent/H_2_O/base) proved unsuccessful. Compounds **1‐BAr_2_** are unstable at raised temperatures in the presence of aqueous base as demonstrated by refluxing **1‐B(C_6_F_5_)_2_** (chosen to facilitate in‐situ reaction monitoring by ^19^F NMR spectroscopy) in etherate solvents in the presence of aqueous K_2_CO_3_ or CsF, which resulted in rapid deboronation of **1‐B(C_6_F_5_)_2_** (<15 min) to form **1**. The formation of **1** by protodeboronation of **1‐B(C_6_F_5_)_2_** was indicated by the observed colour change from dark purple to yellow and confirmed by ^1^H NMR spectroscopy on isolated material. In the absence of a base **1‐B(C_6_F_5_)_2_** shows improved stability to ether/H_2_O mixtures as about 50 % was recovered after refluxing in dioxane with 22 equivalents of water for 16 h whereas no **1‐B(C_6_F_5_)_2_** is recovered on heating under analogous conditions in the presence of K_2_CO_3_ or CsF (Scheme 4). This sensitivity to protodeboronation precludes Suzuki Miyaura cross‐coupling under standard conditions. In contrast borylatively fused 2‐phenylpyridyl analogues are robust under aqueous base/raised temperature Suzuki–Miyaura cross‐coupling conditions.7c This disparity is attributed to the weaker BT→B dative bond, relative to the pyridyl→B dative bond, making the former more prone to ring opening by base, the initial step of the protodeboronation pathway.^16^, 4

**Figure 3:**
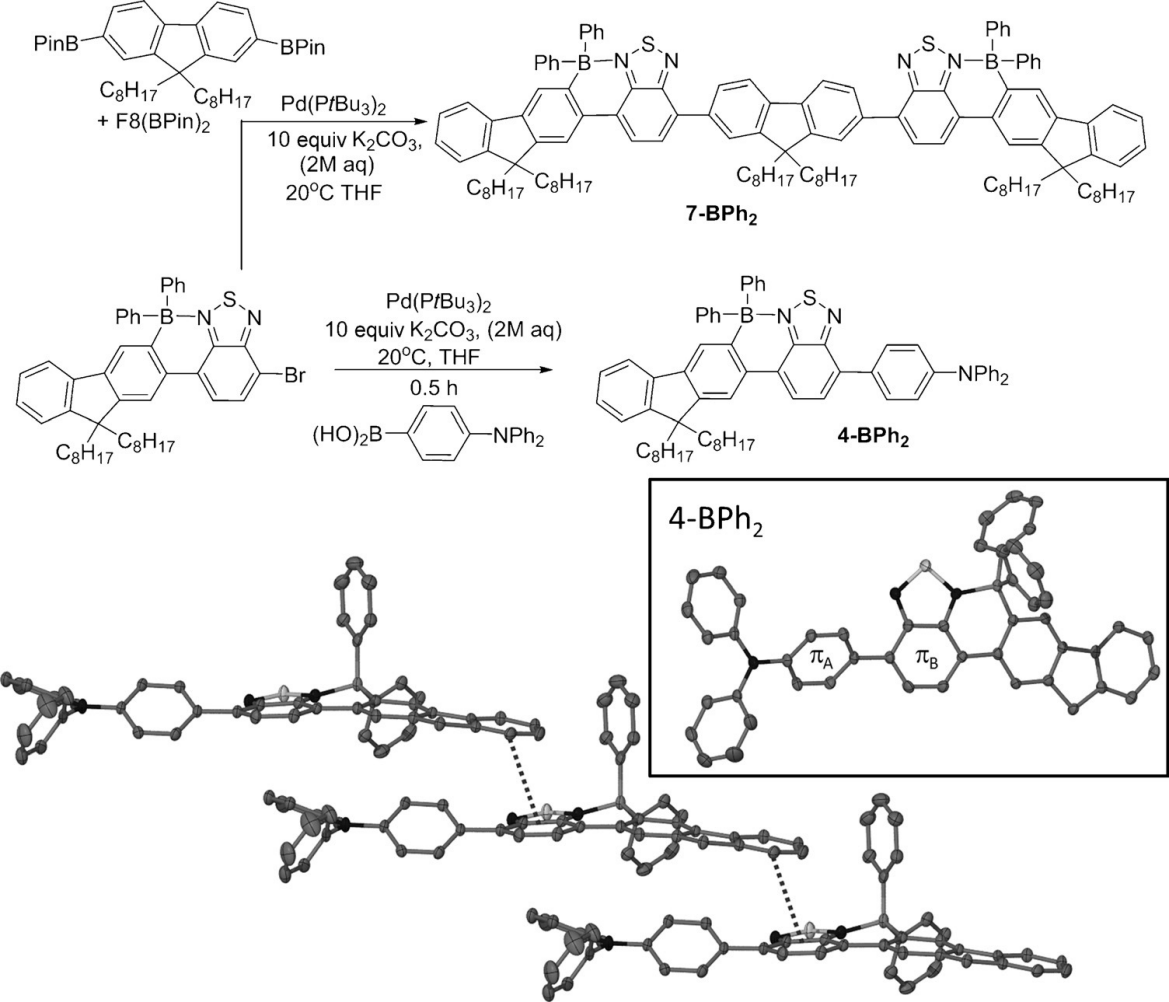
Synthesis of **4‐BPh_2_** and **7‐BPh_2_** by Suzuki–Miyaura cross‐coupling. Bottom: solid state molecular structure (inset) and extended packing structure of **4‐BPh_2_** (hydrogen atoms and the octyl substituents are omitted for clarity), thermal ellipsoids at the 50 % probability level. Dashed contact is the shortest distance (=3.965 Å) between centroids of six membered rings in adjacent molecules.

The successful Suzuki–Miyaura cross‐coupling of **1‐BAr_2_** was achieved by using milder reaction conditions with a highly active catalyst, Pd(P*t*Bu_3_)_2_,^17^ that permitted ambient temperature cross‐coupling in just 30 min. The cross‐coupling of **1‐BPh_2_** with F8(Bpin)_2_ produced **7‐BPh_2_** in an excellent yield (84 %, Figure 3, top). This product represents an alternating F8‐BT oligomer that is borylated exclusively on the peripheral F8 units, this complete regioselectivity would not be accessible through the electrophilic C−H borylation of the parent oligomer, F8‐BT‐F8‐BT‐F8, as a mixture of products would be formed due to competitive borylation on the central F8 unit. The triarylamine appended compound, **4‐BPh_2_**, was also accessible using a similar protocol starting from the boronic acid. Again this borylated isomer would not be accessible by direct electrophilic borylation of the non‐borylated precursor F8‐BT‐C_6_H_4_‐NPh_2_ as electrophilic C−H borylation of triarylamines is documented to be more facile than that of fluorene moieties.^8^ Compound **4‐BPh_2_** was amenable to crystallisation, revealing a nonplanar aromatic backbone. The angle between the N‐C_6_H_4_ and BT rings of 36.5 ° (π_A_–π_B_, respectively, Figure 3, inset) is extremely close to that calculated for **4′‐BPh_2_**. The other structural metrics involving the fused BT‐F8 core are unremarkable and comparable to structures previously reported.^8^ Examination of the extended packing structure of **4‐BPh_2_** revealed that the presence of only two quaternary centres in **4‐BPh_2_** now permits a close π‐stacking interaction involving the conjugated core, with distances between centroids of the π‐systems of adjacent molecules <4 Å (Figure 3, bottom).3

### Electrochemical and photophysical properties

The photophysical properties of **3‐BPh_2_**, **4‐BPh_2_**, **5‐BPh_2_** and **7‐BPh_2_** were investigated by solution UV/Vis absorption and photoluminescence (PL) spectroscopy in toluene (Table 1). For comparison purposes data are included for the borylated F8‐BT‐F8 analogue, **2‐BPh_2_**, previously reported.^8^ Compound **5‐B(C_6_F_5_)_2_** is effectively non‐emissive therefore the photophysical properties are not discussed herein (see the Supporting Information for UV/Vis absorption data for this compound). Toluene was utilised as the solvent for these measurements as in previous studies the photophysical properties of the borylated materials in this solvent most closely match those observed when the materials were dispersed in poly[(9,9‐di‐*n*‐octylfluorenyl‐2,7‐diyl)‐*alt*‐(benzo[2,1,3]‐thiadiazol‐4,8‐diyl)] (PF8‐BT) used for solid state emission studies.^8^, 1

**Table 1:** Comparison of photophysical properties of **2‐BPh_2_**, **3‐BPh_2_**, **4‐BPh_2_**, **5‐BPh_2_** and **7‐BPh_2_** (1×10^−5^ m toluene solutions).

Cpd	*λ*_max abs_ [nm]	*ϵ* [m ^−1^ cm^−1^]	Optical band‐gap [eV]^[a]^	*λ*_max em_ [nm]	Stokes shift [nm]	*Φ* [%]^[b]^
**2‐BPh_2_**	559	12 400	1.92	702	143	10.0
**3‐BPh_2_**	583	12 400	1.83	721	137	1.8
**4‐BPh_2_**	584	13 900	1.81	753	169	0.9
**5‐BPh_2_**	661	44 500	1.68	765	104	1.5
**7‐BPh_2_**	573	34 400	1.91	704	131	12.6

[a] Band‐gap estimated from onset of absorption. [b] Quantum yield estimated by using cresyl violet as standard (*Φ*_**f**_=54 % in methanol)^20^ PLQY error is ±4.5 % efficiency.

Relative to the fluorene‐substituted analogue (**2‐BPh_2_**) compounds **3‐BPh_2_** and **4‐BPh_2_** showed a red‐shifted absorbance (24 and 25 nm, respectively) and emission (19 and 51 nm, respectively). This is consistent with the calculated increase in the HOMO energy level on replacement of the moderately π‐electron donating fluorene unit with the more electron‐rich 2‐methyl‐thiophene or Ph_3_N units. The significantly greater Stokes shift for **4‐BPh_2_** is indicative of greater ICT character consistent with the calculated HOMO and LUMO being more localised on separate parts of the molecule in **4′‐BPh_2_**. Analysis of the more extended structures revealed a substantial decrease in the optical band‐gap and red‐shift in the absorbance (with significant absorbance now in the NIR region of the spectrum)^12^ and emission for **5‐BPh_2_**. The large decrease in optical band‐gap is attributed to a significant increase in the HOMO energy level due to the thieno[3,2‐*b*]thiophene π‐electron bridge maintaining good planarity in **5‐BPh_2_** (by analogy to the effectively planar structure of **5‐B(C_6_F_5_)_2_**) and increasing the effective conjugation length of this molecule. These observations are consistent with literature reports of using thieno[3,2‐*b*]thiophene as an effective π‐electron bridging unit11b and is further supported by cyclic voltammetry measurements (see subsequent discussion). Linking the borylated F8‐BT units with an F8 unit in **7‐BPh_2_** leads to a red‐shift in the *λ*_max abs_ relative to **2‐BPh_2_** but essentially identical emission is observed due to a smaller Stokes shift. The absorbance and emission of **7‐BPh_2_** is blue‐shifted relative to **5‐BPh_2_** which is attributed to two factors: 1) a reduced influence on the HOMO energy level as the fluorene unit is less electron‐rich compared to thieno[3,2‐*b*]thiophene, and 2) increased dihedral angles between the bridging F8 unit and the two borylated F8‐BT units (based on the calculated structure of **2′‐BPh_2_** where these dihedral angles are 39–40 °). The toluene solution PLQY values of the longer wavelength emitting systems **3‐BPh_2_**, **4‐BPh_2_** and **5‐BPh_2_** are relatively low (<2 %) compared to the related fluorine‐substituted systems (≥10 %; **2‐BPh_2_** and **7‐BPh_2_**). This could be due to a number of factors such as the energy‐gap law in which the quantum yield of a fluorophore decreases with a reduction in band‐gap.^3^

**Figure 4:**
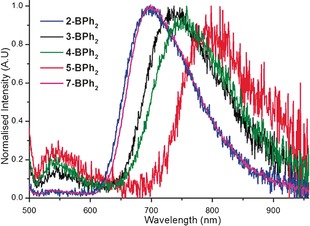
Emission from thin films (films comprising of a 5 wt. % of the relative guest compound dispersed in PF8‐BT).

The emission properties of **3‐BPh_2_**, **4‐BPh_2_**, **5‐BPh_2_** and **7‐BPh_2_** were investigated in thin films prepared from a 5 wt. % mixture of the compounds dispersed in PF8‐BT (a common ambipolar OLED host material) spin‐coated from toluene. On excitation at 468 nm in the PF8‐BT polymer host compounds **3‐BPh_2_** and **4‐BPh_2_** emitted with a *λ*_max_ of 737 and 749 nm, respectively, and excellent (for deep red/near IR emitters) solid state quantum yields of 8.8 and 8.9 %, respectively. In both cases there was only a minor contribution to the overall emission from the PF8‐BT host centred at 550 nm (Figure 4 and Table 2). The emission maxima of **3‐BPh_2_** and **4‐BPh_2_** are significantly red‐shifted compared to the previously reported fluorene analogue **2‐BPh_2_** (data included in Table 2 for comparison purposes).^8^ In the PF8‐BT host the extended compound **7‐BPh_2_** emission is centred at 702 nm, slightly red‐shifted compared to that observed for **2‐BPh_2_**, with a quantum yield of 37.4 % which is excellent for solid‐state emission in this region of the spectrum. No significant emission is observed from the PF8‐BT host with **7‐BPh_2_**. The thieno[3,2,b]thiophene analogue, **5‐BPh_2_**, emits with a considerably red‐shifted *λ*_max_ of 789 nm with appreciable emission up to 950 nm. The quantum yield of 4.4 % (excluding minor emission from the PF8‐BT host) is again excellent for solid state emission in this region of the spectrum. The reduction in quantum yield for **5‐BPh_2_** relative to **3‐BPh_2_** and **4‐BPh_2_** is attributed to the energy gap law^3^ and to the emission of the PF8‐BT host being less well matched with the significantly red‐shifted absorption band of **5‐BPh_2_** resulting in less efficient Förster energy transfer to this guest compound from the host.^18^, 4, 2

**Table 2:** Thin film emission (films comprising of a 5 wt. % of the relative guest compound dispersed in PF8‐BT).

Film	*λ*_max em_ [nm]	*Φ*_f_ [%]^[c]^
PF8‐BT: **2‐BPh_2_** ^[a,b]^	696	32.9 (32.9)
PF8‐BT: **3‐BPh_2_** ^[a,b]^	737	8.8 (9.2)
PF8‐BT: **4‐BPh_2_** ^[a,b]^	749	8.9 (9.7)
PF8‐BT: **5‐BPh_2_** ^[a,b]^	789	4.4 (4.7)
PF8‐BT: **7‐BPh_2_** ^[a,b]^	702	37.4 (37.4)

[a] 95:5 wt %. [b] Excited at 468 nm. [c] Absolute value measured using an intergrating sphere, *Φ*_f_ excluding emission from the PF8‐BT host centred at approximately 550 nm (in parentheses total PLQY). PLQY error is ±4.5 % efficiency.

The observation of significant emission centred at 789 nm from **5‐BPh_2_** and also the intense emission for **7‐BPh_2_** centred at 702 nm is notable as emission from organic chromophores with *λ*_max_>700 nm generally occurs with significantly lower quantum yields even when doped into host materials.^2^ The rigid nature of **5‐BPh_2_** and **7‐BPh_2_** coupled with the four and five, respectively, quaternary centres are presumably important for reducing non‐radiative decay pathways in the solid state. Furthermore, the significant increase in quantum yield upon dispersion in PF8‐BT relative to that in dilute solution may also be due to interactions between the borylated compound and the host polymer that reduces non‐radiative pathways such as rotation/vibration of the exocyclic BPh_2_ groups as observed in borylated compounds that display aggregation induced emission.^4^ To the best of our knowledge the quantum yield from **5‐BPh_2_** dispersed in PF8‐BT is among the best reported for organic chromophores with solid state emission centred around 789 nm.^2^, ^19^

Cyclic voltammetry was employed in order to gain further insight into the effect of different peripheral/bridging aromatic groups on the frontier molecular orbital energies. Compounds **3‐BPh_2_**, **4‐BPh_2_** and **7‐BPh_2_** show a single fully reversible reduction process which is stable for at least 10 cycles (at 50 mV s^−1^), whereas **5‐BPh_2_** begins to show a second irreversible reduction peak at the limit of the potential sweep range of DCM. All four compounds show multiple irreversible oxidation peaks. Cyclic voltammetry (Table 3) revealed the anticipated increase in the HOMO energy level of compound **3‐BPh_2_** (+0.11 eV) and **4‐BPh_2_** (+0.35 eV) relative to that measured for **2‐BPh_2_**. This is consistent with incorporation of the electron‐rich 2‐methylthiophene/triphenylamine unit into the conjugated backbone and is in excellent agreement with the computational results. A further increase in the HOMO energy level is observed for **5‐BPh_2_** (+0.21 eV relative to **2‐BPh_2_**) which is attributed to the aforementioned thieno[3,2‐*b*]thiophene bridging unit extending the effective conjugation length. The measured HOMO energy level of the fluorene unit bridged compound **7‐BPh_2_** is slightly raised (+0.07 eV) relative to **2‐BPh_2_**. The increase in the energy of the HOMO is considerably lower than that observed in the analogous thieno[3,2‐*b*]thiophene‐bridged compound, **5‐BPh_2_**, due to the less electron‐rich nature of fluorene compared to thieno[3,2‐*b*]thiophene in addition to the more sterically encumbered fluorene unit presumably having an increased torsion angle between the peripheral borylated units and the central π‐conjugated F3 bridge unit as previously discussed.

**Table 3:** Comparison of redox properties.

Cpd	*E*_ox_^onset^ [V]^[a]^	*E*_red_^onset^ [V]^[a]^	HOMO [eV]^[a]^	LUMO [eV]^[a]^	Band‐gap [eV]
**2‐BPh_2_**	0.80	−1.28	−6.19	−4.11	2.08
**3‐BPh_2_**	0.69	−1.23	−6.08	−4.16	1.92
**4‐BPh_2_**	0.45	−1.29	−5.84	−4.10	1.74
**5‐BPh_2_**	0.59	−1.18	−5.98	−4.21	1.77
**5‐B(C_6_F_5_)_2_**	0.63	−0.95	−6.02	−4.44	1.58
**7‐BPh_2_**	0.73	−1.29	−6.12	−4.10	2.02

Measured in DCM, (1 mm), with [*n*Bu_4_N][PF_6_] (0.1 m) as the supporting electrolyte at a scan rate of 50 mV s^−1^, potentials are given relative to Fc/Fc^+^ redox couple which is taken to be 5.39 eV below vacuum.^20^

Due to the more localised nature of the LUMO in these systems (Figure 1) the effect of incorporating different peripheral/bridging aromatic groups on the reduction potential is more subtle. Compounds **4‐BPh_2_** and **7‐BPh_2_** have essentially the same LUMO energy level as **2‐BPh_2_** whereas a minor stabilisation of the LUMO energy levels is observed for the thienyl incorporated compounds **3‐BPh_2_** (−0.05 eV) and **5‐BPh_2_** (−0.10 eV). The modest stabilisation of the LUMO observed on replacing a fluorene unit for a thienyl unit is in strong agreement with the trends observed through computational analysis and is attributed to the more planar thienyl based structures leading to a degree of delocalisation of the LUMO. Comparison of **5‐BPh_2_** and **5‐B(C_6_F_5_)_2_** was performed to probe the effect on frontier molecular orbital energies of installing strongly electron‐withdrawing groups onto the boron centre in these extended structures. As previously observed for related compounds^8^ exchanging C_6_H_5_ for C_6_F_5_ groups results in minimal change to the HOMO energy level but a notable decrease in the LUMO energy level (Δ*E* of 0.15 eV from **5‐BPh_2_** to **5‐B(C_6_F_5_)_2_**). Thus **5‐B(C_6_F_5_)_2_** has a low band‐gap and an extremely low lying LUMO at −4.44 eV.

## Conclusions

The readily accessible bromine‐functionalised borylated compounds **1‐BAr_2_** are versatile precursors to extended D–A systems as they are amenable to Stille, Suzuki–Miyaura and Negishi cross‐coupling reactions. This chemistry represents a facile method to synthesise many different borylated D_1_–BT–D_2_ systems, that are selectively borylated on the less nucleophilic aromatic ring and opens up the modular synthesis of a wide array of emissive oligomeric D–A structures. Extended conjugated molecules accessible from this methodology have large Stokes shifts, extremely low LUMO energies, small band‐gaps and significant absorbance and emission in solution and when dispersed in a host polymer, with emission in the far‐red and NIR region of the spectrum important for bioimaging applications.^21^ One example, **5‐BPh_2_**, has significant fluorescence emission centred at 789 nm a notable property that is still challenging to achieve in the solid state.^2^ Related oligomers and polymers are currently under exploration for a range of applications.

## Experimental Section

Unless otherwise indicated all reagents were purchased from commercial sources and were used without further purification. The zincate,^14^ 4‐bromo‐7‐(9,9‐dioctyl‐9*H*‐fluoren‐2‐yl)benzo[*c*][1,2,5]thiadiazole,^22^ Pd(*t*Bu_3_P)_2_,^23^ **2‐BPh_2_** 8 bis(tri‐*n*‐butylstannyl)thieno[3,2‐*b*]thiophene^24^ were synthesised by modified literature procedures. All appropriate manipulations were performed by using standard Schlenk techniques or in an argon‐filled MBraun glovebox (O_2_ levels below 0.5 ppm). Glassware was dried in a hot oven, overnight, and heated under vacuum before use. Solvents and amines were distilled from NaK, CaH_2_, or K and degassed prior to use. ^1^H NMR and ^13^C NMR spectra were recorded using 400 and 500 MHz spectrometers with chemical shift values being reported in ppm relative to residual protio solvent (e.g., in CHCl_3_ in CDCl_3_ *δ*_H_=7.27 or *δ*_C_=77.2 ppm) as internal standards. All coupling constants (*J*) are reported in Hertz (Hz). Other NMR spectra were recorded with a 400 MHz Bruker AV‐400 spectrometer (^11^B; 162 MHz, ^27^Al 104.3 MHz). The ^19^F NMR spectra were referenced to C_6_F_6_, ^11^B NMR spectra were referenced to external BF_3_/Et_2_O, and ^27^Al to Al(NO_3_)_2_ in D_2_O (Al(D_2_O)_6_^3+^). Unless otherwise stated all NMR spectra were recorded at 293 K. Carbon atoms directly bonded to boron are not always observed in the ^13^C{^1^H} NMR spectra due to quadrupolar relaxation leading to signal broadening. All UV/Vis absorption spectra were recorded on a Varian Cary 5000 UV/Vis/NIR spectrophotometer and the solution emission spectra were recorded on a Varian Cary Eclipse fluorometer at room temperature in spectroscopic grade solvents exciting at the relative absorbance maxima. Solid state fluorescence and absolute quantum yields were measured on spin coated films of polymer host/5 wt % emitter using a Hamamatsu C9920–02 Absolute quantum yield measurement system. Cyclic voltammetry was performed using a CH‐Instrument 1110C electrochemical/analyser potentiostat under a nitrogen flow. Measurements were made using a 0.001 m analyte solution with 0.1 m tetrabutylammonium hexafluorophosphate (Fluka, ≥99.0 %) as the supporting electrolyte in dichloromethane that had been degassed prior to use and obtained from a dry solvent system. A glassy carbon electrode served as the working electrode and a platinum wire as the counter electrode. An Ag/AgNO_3_ non‐aqueous reference electrode was used. All scans were calibrated against the ferrocene/ferrocenium (Fc/Fc^+^) redox couple, which in this work is taken to be 5.39 eV below vacuum.^20^ The half‐wave potential of the ferrocene/ferrocenium (Fc/Fc^+^) redox couple (*E*_1/2, Fc,Fc+_) was estimated from *E*_1/2, Fc,Fc+_=(*E*_ap_+*E*_cp_)/2, where *E*_ap_ and *E*_cp_ are the anodic and cathodic peak potentials, respectively. Calculations were performed using the Gaussian 09 suite of programmes.^25^ Structures were pre‐optimised at the DFT B3LYP/6–31G level followed by optimisation at the M06–2X/6–311G+(d,p) level with inclusion of a PCM model for solvent correction (DCM).^26^ Structures were confirmed as minima by frequency analysis and the absence of imaginary frequencies. X‐ray data for compound **4‐BPh_2_** were collected using Mo_Kα_ radiation on an Agilent Supernova, equipped with an Oxford Cryosystems Cobra nitrogen flow gas system. The data were measured using the CrysAlisPro^27^ suite of programs. All structures were solved using direct methods^28^ and refined against *F* ^2^ using the Crystals^29^ software package. Non‐hydrogen atoms were refined anisotropically. Hydrogen atoms were all located in a difference map and repositioned geometrically. Synchrotron X‐ray data were collected at beamline I19 (*λ*=0.6889 Å) Diamond Light Source^30^ for compound **5‐B(C_6_F_5_)_2_**. The data were measured using the GDA suite of programs. The data for **4‐BPh_2_** and **5‐B(C_6_F_5_)_2_** were processed and reduced using the CrysAlisPro^27^ suite of programs. Absorption correction was performed using empirical methods (SADABS) based upon symmetry‐equivalent reflections combined with measurements at different azimuthal angles.^31^, ^32^ All the atoms were refined anisotropically. Hydrogen atoms were placed in calculated positions refined using idealised geometries (riding model) and assigned fixed isotropic displacement parameters. The structure was solved and refined against all *F* ^2^ values using the SHELXTL and Olex 2 suite of programs.^33^ Crystallographic details are given in the Supporting Information.

CCDC 1443404 (**8‐B(C_6_F_5_)_2_**), 1455079 (**5‐B(C_6_F_5_)_2_**) and 1457709 (**4‐BPh_2_**) contain the supplementary crystallographic data for this paper. These data are provided free of charge by The Cambridge Crystallographic Data Centre.

### Synthesis and characterisation

**Synthesis of 1‐BPh_2_**: BCl_3_ (1 m solution) in DCM (5 mL, 5 mmol) was added to a bright yellow solution of **1** (500 mg, 0.83 mmol) in DCM (10 mL). The reaction mixture was stirred for 16 h under the flow of nitrogen whereupon the colour changed from yellow to dark purple was observed. The solvent and unreacted BCl_3_ were removed under reduced pressure to yield a dark purple residue. The reaction mixture was then dissolved in toluene (10 mL) and ZnPh_2_ (400 mg, 1.82 mmol) was added to the reaction mixture. The reaction mixture was stirred for 2 h, “wet” toluene (unpurified toluene used as received; 10 mL) was added to the reaction mixture which was then passed through a plug of silica. The resulting solution was purified by silica‐gel chromatography (5 % NEt_3_ in hexane; eluent: hexane/DCM 9:1) to afford the desired product as a dark purple residue (yield 456 mg, 72 %). ^1^H NMR (400 MHz, CDCl_3_): *δ*=8.21 (d, *J*=7.8 Hz, 1 H), 8.08 (d, *J*=7.8 Hz, 1 H), 8.03 (s, 1 H), 7.83 (s, 1 H), 7.68–7.59 (m, 1 H), 7.41–7.15 (m, 13 H), 2.05 (t, *J*=8.3 Hz, 4 H), 1.28–1.00 (m, 20 H), 0.90–0.61 ppm (m, 10 H); ^13^C{^1^H} NMR (101 MHz, CDCl_3_): *δ*=154.3, 153.0, 152.2, 151.3, 149.1, 147.8, 142.4, 140.7, 135.3, 133.6, 130.1, 128.9, 127.6, 127.4, 126.7, 126.1, 125.8, 123.5, 122.7, 120.6, 116.4, 110.5, 54.8, 40.6, 31.7, 30.0, 29.2, 29.1, 23.9, 22.5, 14.0 ppm; ^11^B NMR (128 MHz, CDCl_3_): *δ*=2.8 ppm (br); HRMS (APCI) calcd for C_47_H_53_BBrN_2_S^+^ [*M*−H]^+^ 767.3200, found 767.3197.

**Synthesis of 1‐B(C_6_F_5_)_2_**: BCl_3_ (1 m solution) in DCM (3.3 mL, 3.3 mmol) was added to a bright yellow solution of 1 (330 mg, 0.55 mmol) in DCM (10 mL) in a Schlenk flask. The reaction mixture was stirred for 16 h during which time a colour change from yellow to dark purple was observed. The solvent and any reacted BCl_3_ were removed under reduced pressure to yield a dark purple residue. The reaction mixture was then dissolved in DCM (10 mL) and Zn(C_6_F_5_)_2_ (484 mg, 0.12 mmol) was added. The reaction mixture was stirred for 2 h and then after the addition of wet DCM the solution was passed through a plug of silica. The solvent was removed under reduced pressure to afford a dark purple residue (yield 497 mg, 96 %). ^1^H NMR (400 MHz, CD_2_Cl_2_): *δ*=8.32 (d, *J*=7.9 Hz, 1 H), 8.17 (d, *J*=7.8 Hz, 1 H), 8.05 (s, 1 H), 7.76 (s, 1 H), 7.62–7.69 (m, 1 H), 7.25–7.41 (m, 3 H), 1.95–2.13 (m, 4 H), 1.00–1.22 (m, 20 H), 0.78 (t, *J*=7.0 Hz, 6 H), 0.58–0.74 ppm (m, 4 H); ^13^C{^1^H} NMR (101 MHz, CD_2_Cl_2_): *δ*=153.7, 152.0, 151.0, 148.0, 143.9, 140.8, 136.5, 129.6, 128.4, 128.2, 127.4, 126.3, 124.8, 123.6, 120.8, 117.3, 112.0, 55.6, 41.0, 32.3, 30.5, 29.8, 29.7, 24.4, 23.1, 14.4 ppm; ^11^B NMR (128 MHz, CD_2_Cl_2_): *δ*=−5.0 ppm (br); ^19^F NMR (376 MHz, CD_2_Cl_2_): *δ*=−131.91 (dd, *J*=20.2, 8.3 Hz, 4 F), −157.50 (d, *J*=20.3 Hz, 2 F), −163.47 ppm (m, 4 F); HRMS (APCI) calcd for C_47_H_53_BBrN_2_S^+^ [*M*+K]^+^: 985.1817, found 985.2341.

**Synthesis of 3‐BPh_2_**: **1‐BPh_2_** (120 mg, 0.15 mmol), tri‐*n*‐butyl(5‐methylthiophen‐2‐yl)stannane (75 mg, 0.195 mmol) and Pd(PPh_3_)_4_ were dissolved in THF (10 mL). The reaction mixture was heated at 75 °C for 20 h. After being cooled to room temperature the solvent was removed under reduced pressure and the residue was purified by column chromatography on silica gel (eluent: hexane followed by hexane/DCM 8:2) to afford **3‐BPh_2_** as a dark blue residue (yield 96 mg, 81 %). ^1^H NMR (400 MHz, CDCl_3_): *δ*=8.21 (d, *J*=7.8 Hz, 1 H), 7.99 (s, 1 H), 7.91–7.75 (m, 3 H), 7.68–7.57 (m, 1 H), 7.39–7.09 (m, 13 H), 6.80 (dd, *J*=1.0, 3.5 Hz, 1 H), 2.55 (s, 3 H), 2.04 (t, *J*=8.3 Hz, 4 H), 1.24–0.97 (m, 20 H), 0.86–0.63 ppm (m, 10 H); ^13^C{^1^H} NMR (101 MHz, CDCl_3_): *δ*=154.7, 152.0, 151.5, 151.3, 148.9, 148.0, 142.2, 141.9, 140.9, 135.5, 133.7, 129.6, 128.1, 128.1, 127.7, 127.5, 127.1, 126.8, 126.6, 125.9, 125.7, 125.2, 123.8, 122.7, 120.4, 116.0, 77.3, 76.7, 54.7, 40.6, 31.8, 30.1, 29.2, 29.2, 23.9, 22.6, 15.5, 14.1 ppm; ^11^B NMR (128 MHz, CDCl_3_): *δ*=2.3 ppm (br); MALDI‐TOF: calcd for C_46_H_52_BN_2_S_2_^+^ [*M*−C_6_H_5_]^+^: 707.4, found 707.6.

**Synthesis of 4‐BPh_2_**: **1‐BPh_2_** (129 mg, 0.17 mmol), 4‐(diphenylamino)phenylboronic acid (68 mg, 0.24 mmol) and Pd(*t*Bu_3_P)_2_ (13 mg, 0.025 mmol) were dissolved in THF (5 mL). K_3_PO_4_ (2 m aq.; 1 mL, 2 mmol) was added to the reaction mixture which was stirred at room temperature for 30 min. The solution was then diluted with THF (20 mL) and water (30 mL) was added. The reaction mixture was then washed with brine (2×100 mL). The organic layer was isolated and dried (MgSO_4_). The solvent was evaporated under reduced pressure and the residue was purified by column chromatography (eluent: graduated petroleum ether/toluene 9:1 to 6:4) to afford the desired product as a dark purple residue (yield 123 mg, 79 %). ^1^H NMR (400 MHz, CD_2_Cl_2_): *δ*=8.42 (d, *J*=8.0 Hz, 1 H), 8.15 (s, 1 H), 7.95 (d, *J*=7.8 Hz, 1 H), 7.86–7.93 (m, 3 H), 7.63–7.68 (m, 1 H), 7.39–7.43 (m, 1 H), 7.11–7.37 (m, 24 H), 2.05–2.18 (m, 4 H), 1.09–1.26 (m, 20 H), 0.72–0.95 ppm (m, 10 H); ^13^C{^1^H} NMR (101 MHz, CD_2_Cl_2_): *δ*=155.5, 154.2, 152.4, 152.0, 149.7, 149.2, 148.9, 147.8, 142.4, 141.5, 134.2, 132.0, 130.4, 130.4, 130.3, 130.0, 129.3, 128.8, 128.1, 127.8, 127.3, 126.5, 125.8, 125.7, 124.9, 124.3, 123.5, 123.0, 120.8, 117.1, 55.5, 41.2, 32.4, 30.7, 29.8, 29.8, 24.6, 23.2, 14.5 ppm; ^11^B NMR (128 MHz, CDCl_3_): *δ*=2.3 ppm (br); MALDI‐TOF: calcd for C_59_H_61_BN_3_S^+^ [*M*−C_6_H_5_]^+^: 855.0, found 854.7.

**Synthesis of 5‐BPh_2_**: Compound **1‐BPh_2_** (100 mg, 0.13 mmol), 2,5‐bis(trimethylstannyl)thieno[3,2‐*b*]thiophene (28 mg, 0.06 mmol), [Pd_2_(dba)_3_] (96 mg, 0.0065) and S‐Phos (18 mg, 0.013) were dissolved in THF (3 mL). The reaction mixture was heated at 70 °C for 20 h during which time it changed colour from dark purple to dark green. After being cooled to room temperature the solvent was removed under reduced pressure and the residue was purified by column chromatography on base treated silica gel (5 % NEt_3_ in hexane; eluent: hexane followed by hexane/DCM 8:2) to afford **5‐BPh_2_** as a dark green residue (yield 88 mg, 97 %). ^1^H NMR (400 MHz, CD_2_Cl_2_): *δ*=8.40 (s, 2 H), 8.29–8.37 (m, *J*=7.9 Hz, 2 H), 8.12 (s, 2 H), 8.01–8.10 (m, *J*=7.7 Hz, 2 H), 7.85 (s, 2 H), 7.64 (d, *J*=8.1 Hz, 2 H), 7.39 (d, *J*=6.4 Hz, 2 H), 7.14–7.36 (m, 24 H), 1.95–2.23 (m, 8 H), 1.25–1.01 (m, 40 H), 0.64–0.94 ppm (m, 20 H); ^13^C{^1^H} NMR (101 MHz, CDCl_3_): *δ*=155.3, 152.9, 152.7, 152.1, 149.8, 148.8, 142.8, 141.8, 141.7, 141.3, 134.1, 130.2, 129.6, 129.2, 128.2, 128.0, 127.3, 126.6, 125.9, 125.6, 124.6, 123.5, 121.0, 120.9, 117.3, 55.5, 41.1, 32.4, 30.7, 29.8, 29.8, 24.6, 23.2, 14.4 ppm; ^11^B NMR (128 MHz, CDCl_3_): *δ*=No ^11^B NMR resonance was observed at 20 °C HRMS (APCI) calcd for C_100_H_107_B_2_N_4_S_2_^+^ [*M*+H]^+^: 1514.7593, found 1514.7600.

**Synthesis of 5‐B(C_6_F_5_)_2_**: Compound **1‐B(C_6_F_5_)_2_** (150 mg, 0.16 mmol), 2,5‐bis(tri‐*n*‐butylstannyl)thieno[3,2‐*b*]thiophene (56 mg, 0.08 mmol) and Pd(PPh_3_)_4_ (17 mg, 0.016 mmol) was dissolved in toluene (4 mL) and heated at 100 °C for 40 h. After evaporating the solvent, the residue was purified by column chromatography on silica gel (eluent: hexane/DCM 6:4) to afford **5‐B(C_6_F_5_)_2_** as a dark green residue (yield 51 mg, 35 %). ^1^H NMR (400 MHz, CDCl_3_): *δ*=8.53–8.43 (m, 4 H), 8.16 (d, *J*=7.8 Hz, 2 H), 8.03 (s, 2 H), 7.78 (s, 2 H), 7.74–7.65 (m, 2 H), 7.40–7.29 (m, 6 H), 2.06 (t, *J*=8.2 Hz, 8 H), 1.23–1.01 (m, 40 H), 0.85–0.62 ppm (m, 20 H); ^19^F NMR (376 MHz, CDCl_3_): *δ*=−131.52 (dd, *J*=24.1, 8.7 Hz, 8 F), −156.32 (t, *J*=20.3 Hz, 4 F), −162.42 ppm (m, 8 F); MALDI‐TOF: calcd for C_100_H_86_B_2_N_4_S_4_^+^ [*M*]^+^: 1872.6, found 1872.0.

**Synthesis of 6‐BPh_2_**: Compound **1‐BPh_2_** (50 mg, 0.065 mmol), zincate (75 mg, 0.078), [Pd_2_(dba)_3_] (3.5 mg, 0.004 mmol) and S‐Phos (5.3 mg, 0.013 mmol) were dissolved in THF (3 mL). The reaction mixture was heated at 70 °C for 20 h. After being cooled to room temperature the solvent was removed under reduced pressure and the residue was dissolved in hexane and passed through a short plug of silica. A mixture of hexane/DCM (8:2) was then passed through the silica plug and only the purple coloured fractions were collected. Evaporation of the solvent gave the desired product as a dark purple residue (yield 50 mg, 98 %). ^1^H NMR (400 MHz, CDCl_3_): *δ*=8.44 (d, *J*=7.7 Hz, 1 H), 8.10 (s, 1 H), 7.98 (d, *J*=7.5 Hz, 1 H), 7.92–7.82 (m, 3 H), 7.70–7.62 (m, 1 H), 7.46–7.17 (m, 15 H), 2.51 (s, 3 H), 2.09 (t, *J*=7.8 Hz, 4 H), 1.30–1.04 (m, 20 H), 0.91–0.67 ppm (m, 10 H); ^13^C{^1^H} NMR (101 MHz, CDCl_3_): *δ*=154.7, 153.7, 151.8, 151.3, 149.0, 148.3, 142.0, 141.0, 139.0, 133.7, 132.8, 131.8, 130.6, 129.6, 129.2, 128.9, 128.8, 127.5, 127.1, 126.6, 125.9, 125.8, 123.8, 122.8, 120.5, 116.2, 54.8, 40.6, 31.8, 30.1, 29.2, 23.9, 22.6, 21.3, 14.0 ppm; ^11^B NMR (128 MHz, CDCl_3_): *δ*=≈2.0 (br); MALDI‐TOF: calcd for C_48_H_54_BN_2_S_2_^+^ [*M*−C_6_H_5_]^+^: 701.4, found 701.7.

**Synthesis of 7‐BPh_2_**: Compound **1‐BPh_2_** (100 mg, 0.13 mmol), 2,2′‐(9,9‐dioctyl‐9*H*‐fluorene‐2,7‐diyl)bis(4,4,5,5‐tetramethyl‐1,3,2‐dioxaborolane (40 mg, 0.06 mmol) and Pd(*t*Bu_3_P)_2_ (4 mg, 0.007 mmol) were dissolved in THF (3 mL). K_3_PO_4_ (2 m aq.; 0.62 mL, 1.2 mmol) was added to the reaction mixture which was stirred at room temperature for 30 min. The solution was diluted with THF (100 mL) and then washed with brine (100 mL). The organic layer was isolated using a separating funnel and dried (MgSO_4_). The solvent was evaporated under reduced pressure and the residue was purified by column chromatography (eluent: hexane followed by hexane/DCM 8:2) to afford the desired product as a dark purple residue (yield 92 mg, 84 %). ^1^H NMR (400 MHz, CD_2_Cl_2_): *δ*=8.52 (d, *J*=7.9 Hz, 2 H), 8.19 (s, 2 H), 8.14 (d, *J*=7.6 Hz, 2 H), 8.10 (s, 2 H), 8.06–8.01 (m, 2 H), 7.97 (d, *J*=7.9 Hz, 2 H), 7.89 (s, 2 H), 7.68 (dd, *J*=5.6, 1.9 Hz, 2 H), 7.46–7.38 (m, 2 H), 7.37–7.16 (m, 24 H), 2.23–2.04 (m, 12 H), 1.27–1.03 (m, 60 H), 0.93–0.71 ppm (m, 30 H); ^13^C{^1^H} NMR (101 MHz, CD_2_Cl_2_): *δ*=155.5, 154.3, 152.5, 152.0, 149.6, 148.8, 142.5, 141.7, 141.4, 135.4, 134.1, 132.8, 131.4, 130.3, 129.4, 128.6, 128.1, 127.9, 127.2, 126.5, 125.8, 124.8, 124.4, 123.5, 120.9, 120.8, 117.2, 56.1, 55.4, 41.1, 40.7, 32.4, 30.6, 30.5, 29.8, 29.8, 24.6, 23.2, 14.4 ppm; ^11^B NMR (128 MHz, CDCl_3_): *δ*=No ^11^B NMR peak was observed at 20 °C. HRMS (APCI) calcd for C_123_H_145_B_2_N_4_S_2_^+^ [*M*+H]^+^: 1765.1125, found 1765.1135.

## Supporting information

As a service to our authors and readers, this journal provides supporting information supplied by the authors. Such materials are peer reviewed and may be re‐organized for online delivery, but are not copy‐edited or typeset. Technical support issues arising from supporting information (other than missing files) should be addressed to the authors.

Supplementary
