# Supplementary material for: Highly Emissive Far Red/Near‐IR Fluorophores Based on Borylated Fluorene–Benzothiadiazole Donor–Acceptor Materials
Source: Chemistry. 2016 Jul 27;22(35):12439–48. doi: 10.1002/chem.201602010 (PMC6680280; doi:10.1002/chem.201602010)
Supplement: Supplementary file 1 — Supplementary [file CHEM-22-12439-s001.pdf]

CHEMISTRY
A European Journal

Supporting Information

Highly Emissive Far Red/Near-IR Fluorophores Based on Borylated Fluorene–Benzothiadiazole Donor–Acceptor Materials

Daniel L. Crossley,[a] Inigo Vitorica-Yrezabal,[a] Martin J. Humphries,[b] Michael L. Turner,*[a] and Michael J. Ingleson*[a]

chem_201602010_sm_miscellaneous_information.pdf

Contents

Synthetic Procedures	S2
NMR Spectra	S4
UV-vis and Fluorescence Spectra	S20
Cyclic Voltammograms	S25
Crystallographic Details	S26
Optimised Structure Coordinates	S28

Synthetic Procedures

Synthesis of **1**

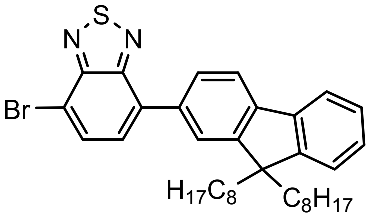

4,7-Dibromobenzo[*c*][1,2,5]thiadiazole (2.64 g, 9 mmol), 9,9-dioctylfluorene-2-boronic acid pinacol ester, (1.55 g, 3 mmol) and $Pd(P^tBu_3)_2$ (0.09 g, 0.18 mmol) were mixed in dry THF (40 mL) under an inert atmosphere. K_3PO_4 (2M aq.) (7.5 mL, 15 mmol) was added to the reaction mixture which was stirred at room temperature for 3 hours. The reaction mixture was diluted with THF (50 mL) and then washed with brine (100 mL). The organic layer was then isolated using a separating funnel and dried over $MgSO_4$. The solvent was evaporated under reduced pressure and a hexane:toluene (8:2) mixture was added to the residue. The residue was filtered and washed with petroleum ether (100 mL). The resulting solid (unreacted 4,7-dibromobenzo[*c*][1,2,5]thiadiazole) was discarded and the filtrate was then evaporated to dryness and the resulting residue was purified by column chromatography on silica gel [eluent: hexane:toluene (8:2)] to afford **1** as a yellow viscous oil. (Yield 1.10 g, 61 %)

^{1}H NMR (400 MHz, CD_2Cl_2) δ = 7.91 - 7.98 (m, 3 H), 7.84 - 7.89 (m, 1 H), 7.76 - 7.82 (m, 1 H), 7.67 (d, *J* = 7.6 Hz, 1 H), 7.44 - 7.33 (m, 3 H), 1.97 - 2.14 (m, 4 H), 1.00 - 1.25 (m, 20 H), 0.80 (t, *J* = 7.0 Hz, 6 H), 0.78 - 0.67 (m, 4 H);

^{13}C{^{1}H} NMR (101 MHz, CD_2Cl_2) δ = 154.5, 153.9, 151.9, 151.7, 142.3, 141.1, 135.9, 135.0, 132.9, 128.8, 128.5, 128.0, 127.5, 124.5, 123.6, 120.6, 120.2, 113.1, 55.8, 40.8, 32.4, 30.6, 29.8, 29.8, 24.5, 23.2, 14.4;

MALDI-TOF: calc. for $C_{35}H_{43}N_2SBr^+$ $[M + H]^+$ = 604.7, found 604.7

Synthesis of **8-B(C_6F_5)$_2$**

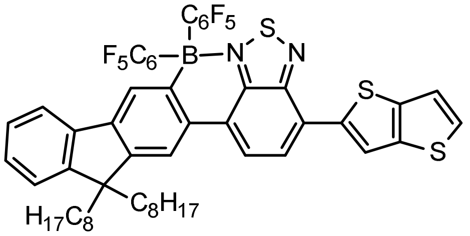

8-B(C_6F_5)$_2$ was isolated as a dark blue solid from the reaction mixture of **5-B(C_6F_5)$_2$**. (Yield 19 mg, 24 %)

^{1}H NMR (400 MHz, $CDCl_3$) δ = 8.46 - 8.36 (m, 2 H), 8.08 (d, *J* = 7.8 Hz, 1 H), 8.02 (s, 1 H), 7.78 (s, 1 H), 7.74 - 7.65 (m, 1 H), 7.51 (d, *J* = 5.3 Hz, 1 H), 7.42 - 7.30 (m, 4 H), 2.06 (t, *J* = 8.2 Hz, 4 H), 1.23 - 1.00 (m, 20 H), 0.85 - 0.56 (m, 10 H);

^{13}C{^{1}H} NMR (101 MHz, $CDCl_3$) δ = 152.0, 151.3, 150.3, 147.7, 143.1, 140.6, 140.3, 140.1, 139.0, 129.5, 128.5, 128.0, 127.9, 127.7, 126.9, 125.9, 125.6, 124.3, 122.9, 121.0, 120.4, 119.6, 116.3, 55.0, 40.6, 31.8, 30.0, 29.2, 29.1, 23.8, 22.5, 14.0;

^{19}F{^{1}H} NMR (376 MHz, $CDCl_3$) δ = -131.51 (dd, *J* = 22.6, 8.7, 4F), -156.46 (t, *J* = 20.30, 2F), -162.49 (m, 4F);

^{11}B NMR (128 MHz, $CDCl_3$) δ = ~-6 (br.);

MALDI-TOF: calc. for $C_{53}H_{45}BF10N_2S_3^+$ $[M]^+$ = 1006.3, found 1006.4

NMR Spectra

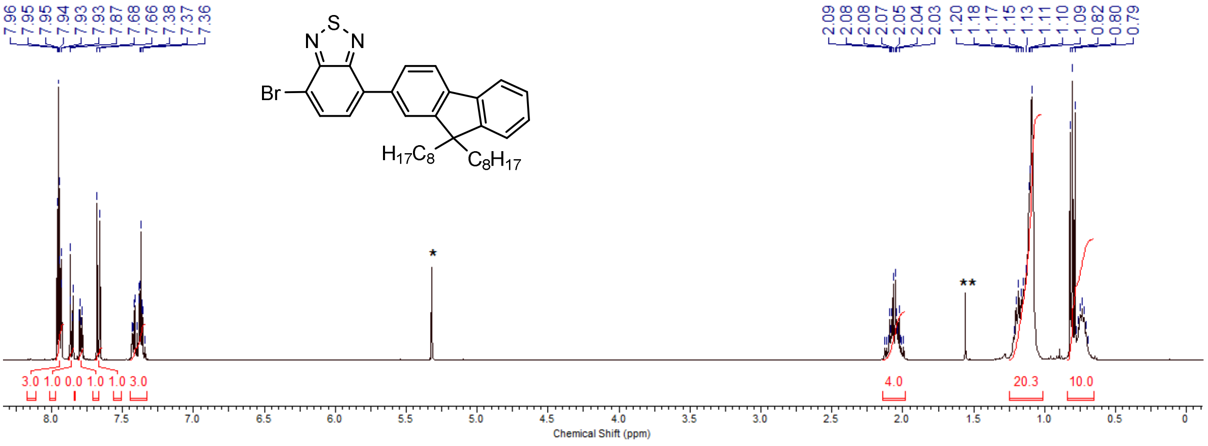

^{1}H NMR spectra of **1** in CD_2Cl_2. * = CH_2Cl_2, ** = H_2O

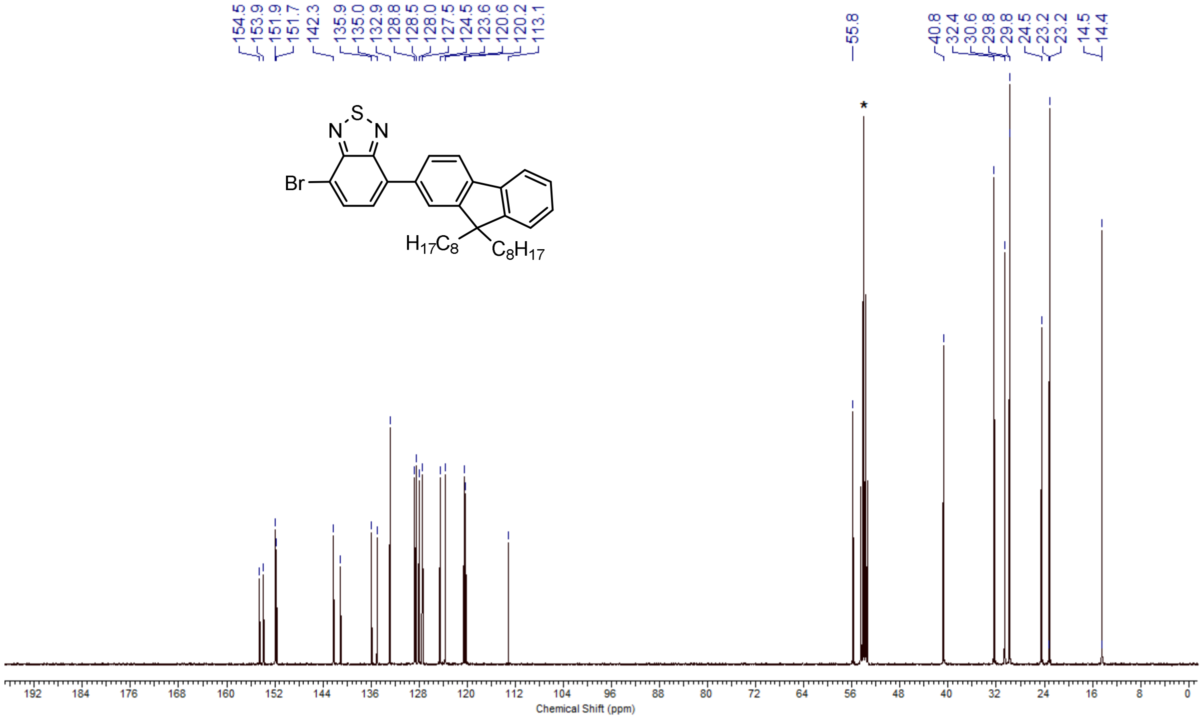

^{13}C{^{1}H} NMR spectra of **1** in CD_2Cl_2. * = CD_2Cl_2

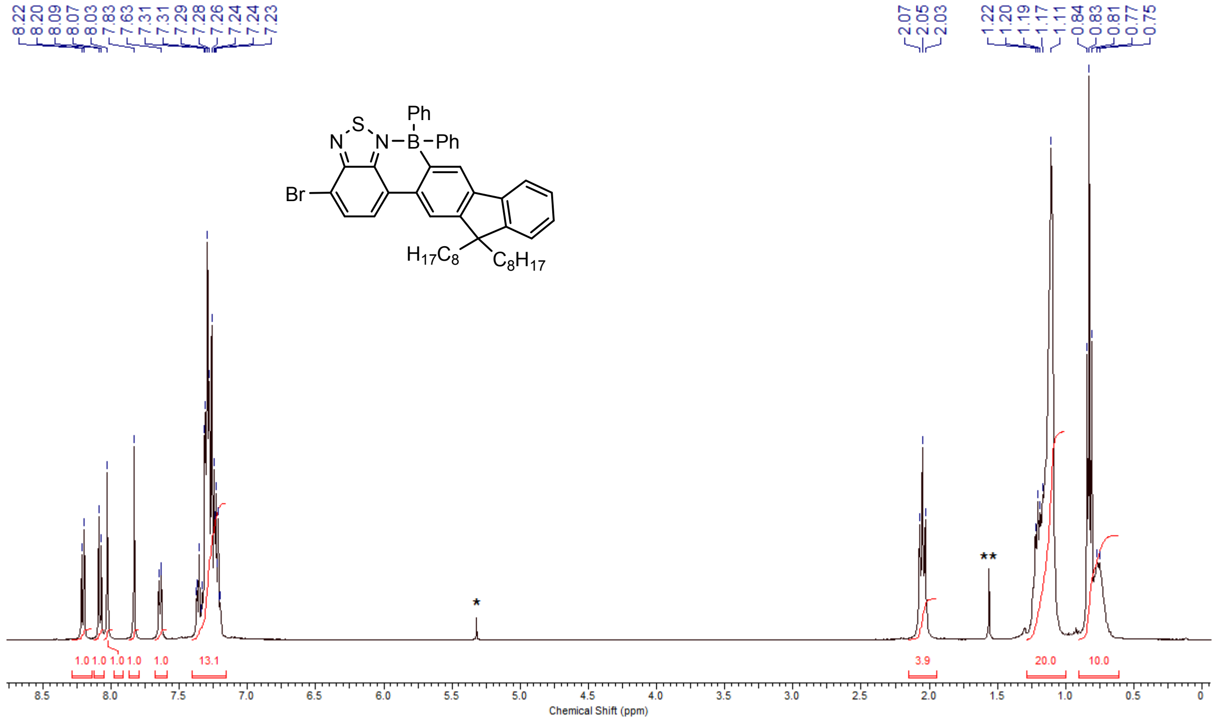

^1H NMR spectra of **1-BPh₂** in $CDCl_3$. * = CH_2Cl_2, ** = H_2O

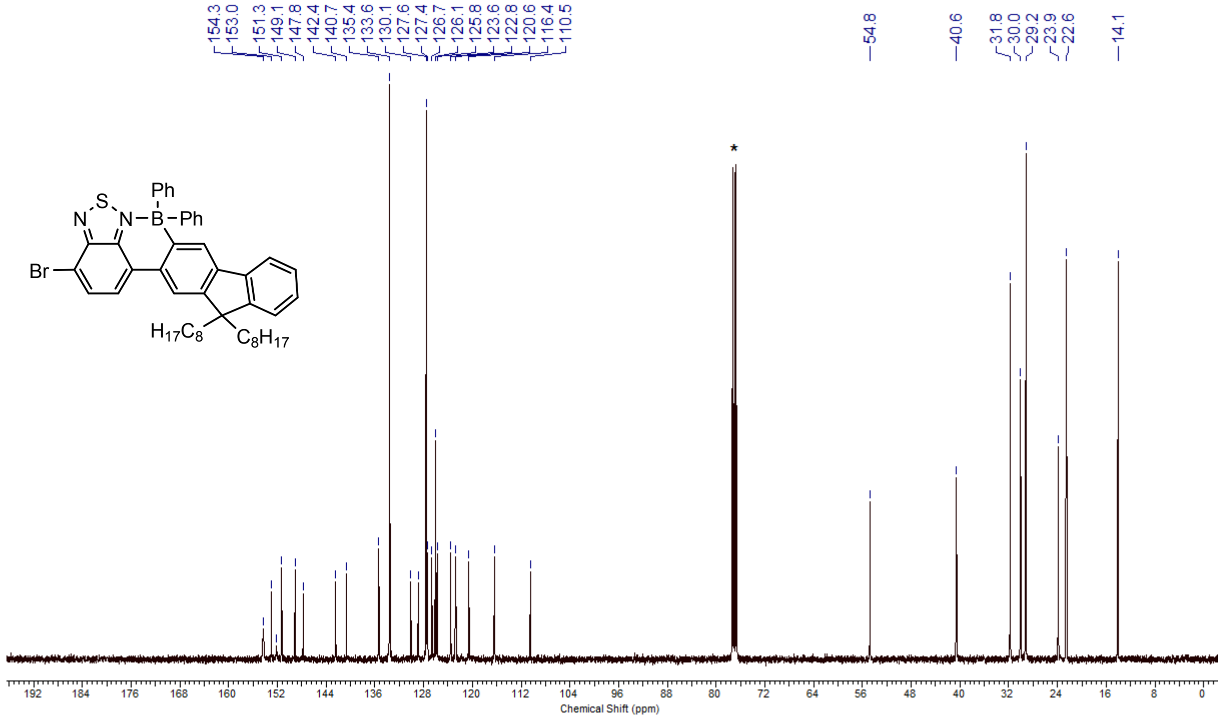

$^{13}C\{^1H\}$ NMR spectra of **1-BPh₂** in $CDCl_3$. * = $CDCl_3$

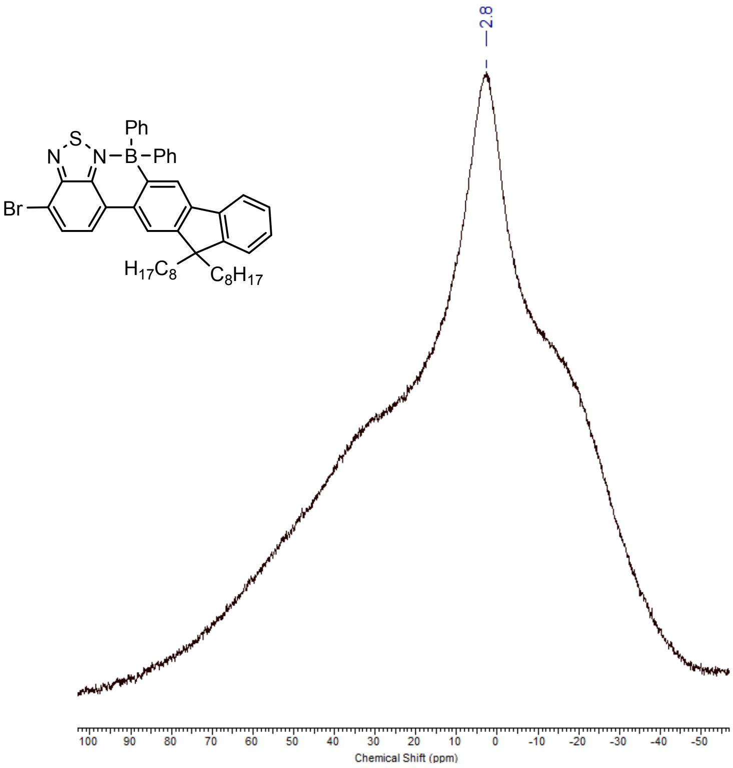

^{11}B NMR spectra of **1-BPh$_2$** in $CDCl_3$.

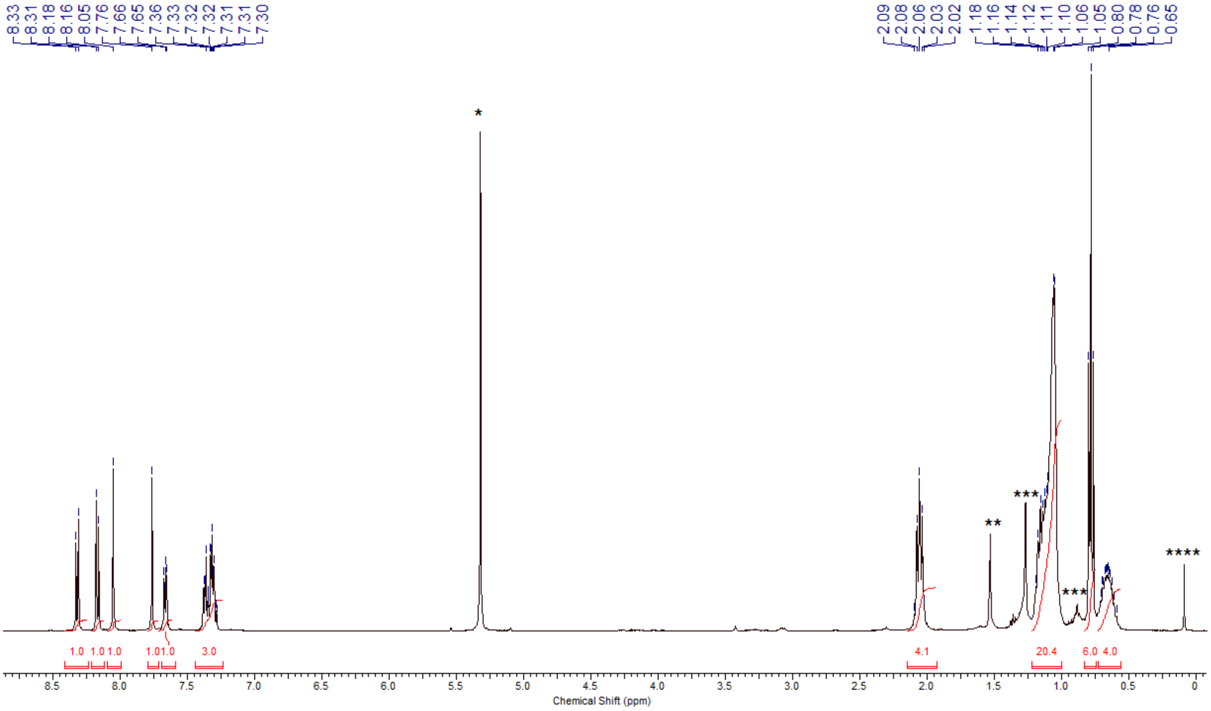

^{1}H NMR spectra of **1-B(C_6F_5)$_2$** in CD_2Cl_2. * = CH_2Cl_2, ** = H_2O, *** = Apiezon H Grease, **** = Silicon Grease.

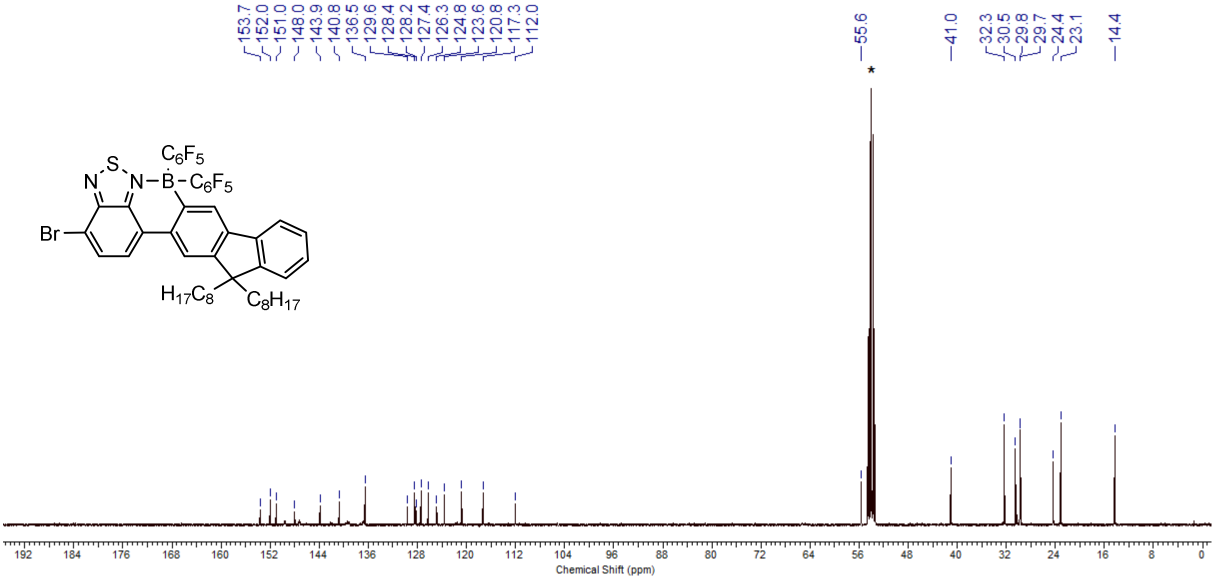

^{13}C{^{1}H} NMR spectra of **1-B(C_6F_5)$_2$** in CD_2Cl_2. * = CH_2Cl_2

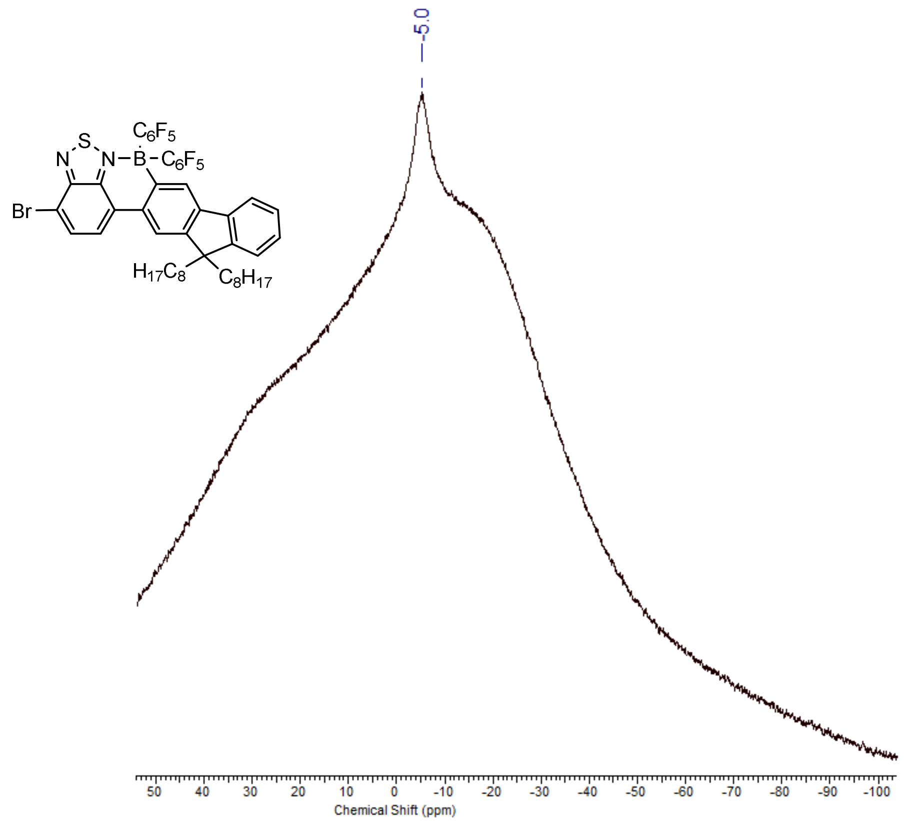

^{11}B NMR spectra of **1-B(C$_6$F$_5$)$_2$** in CD$_2$Cl$_2$.

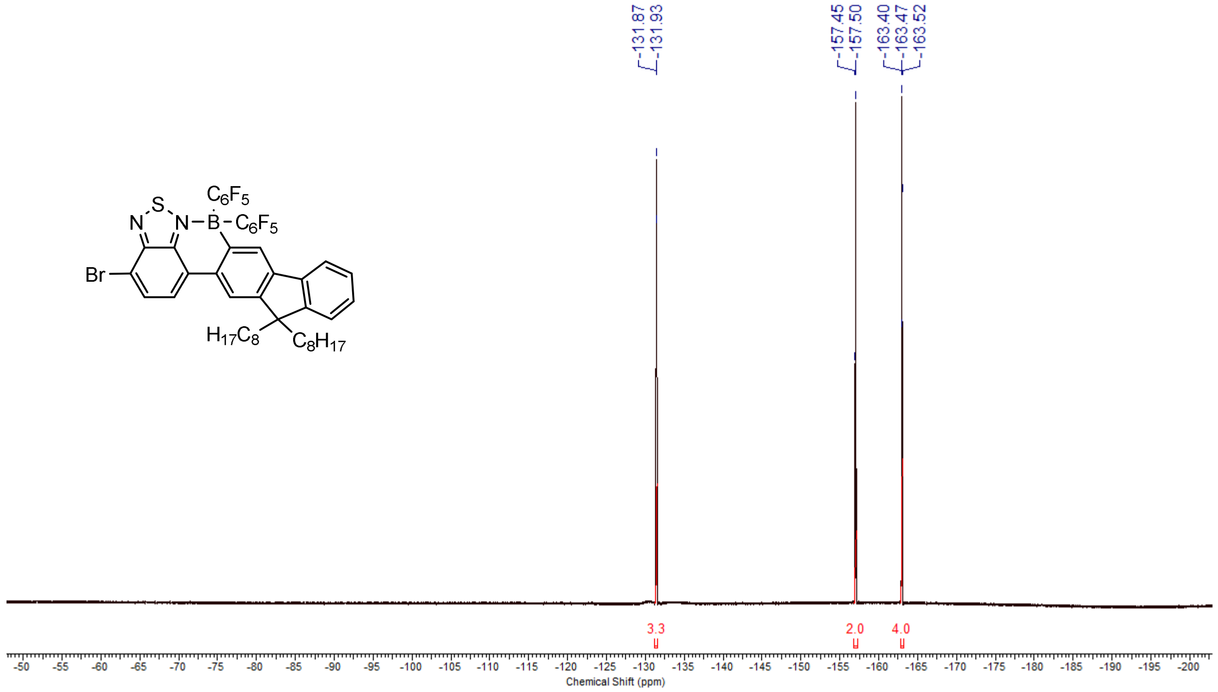

^{19}F NMR spectra of **1-B(C$_6$F$_5$)$_2$** in CD$_2$Cl$_2$.

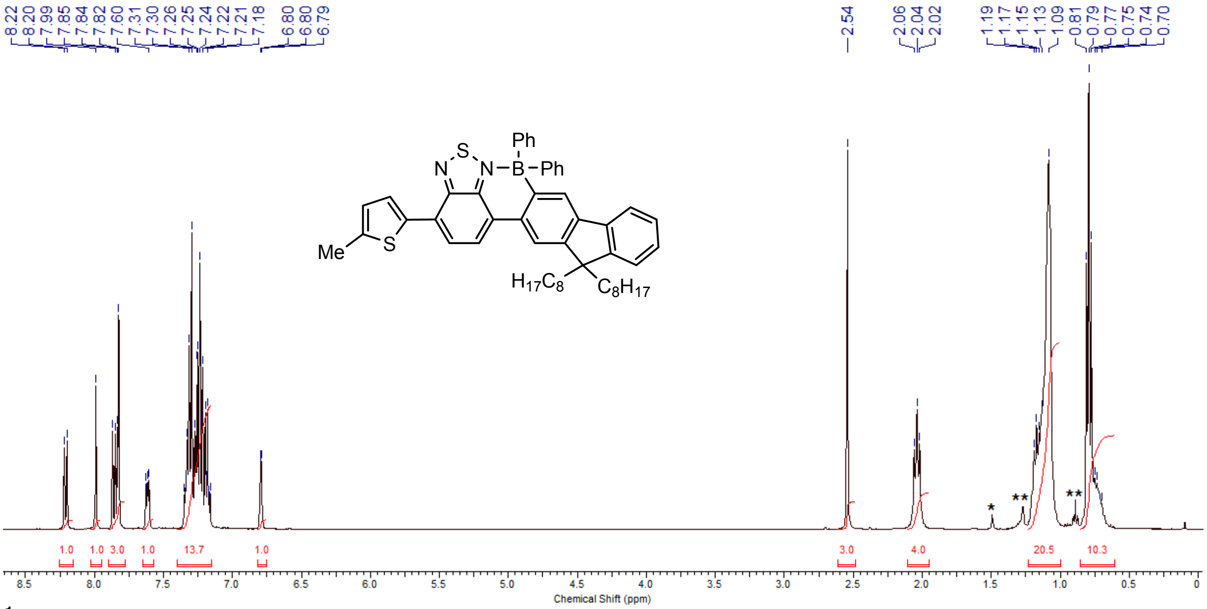

^{1}H NMR spectra of **3-BPh$_2$** in CDCl$_3$. * = H_2O, ** = Hexane

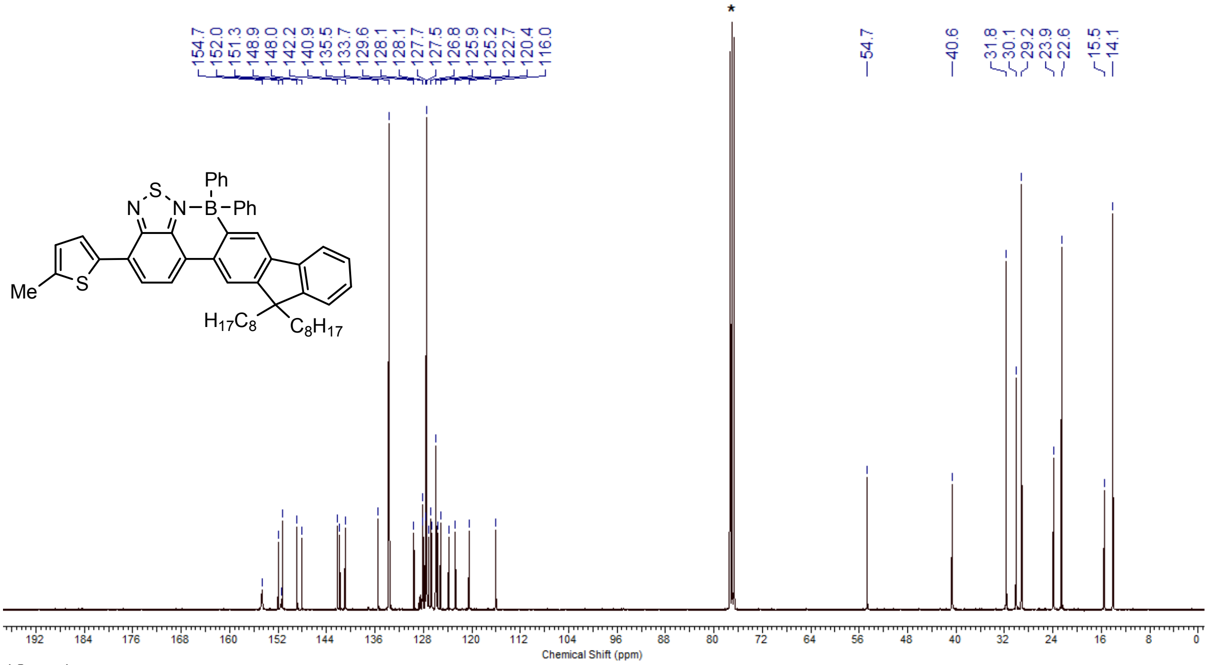

^{13}C{^{1}H} NMR spectra of **1** in CDCl$_3$. * = CDCl$_3$

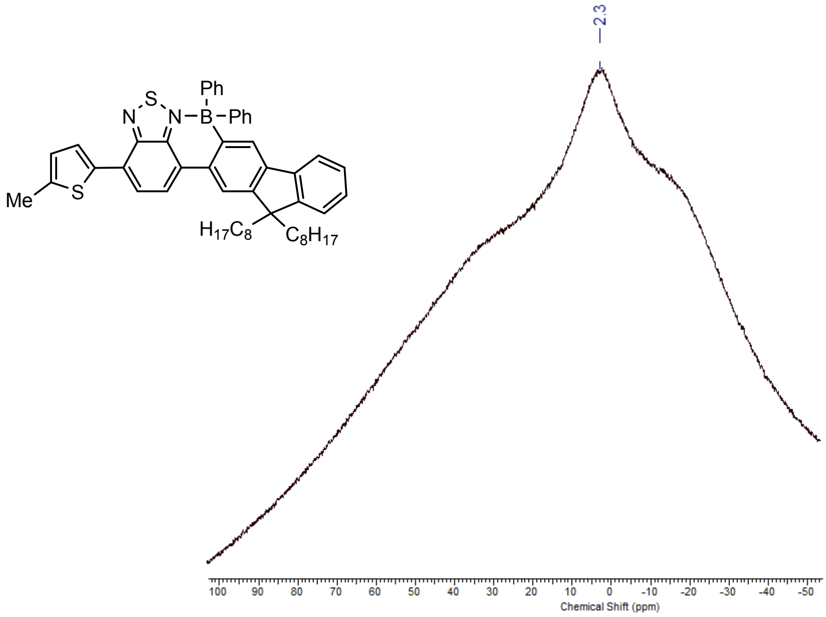

^{11}B NMR spectra of **3-BPh$_2$** in $CDCl_3$.

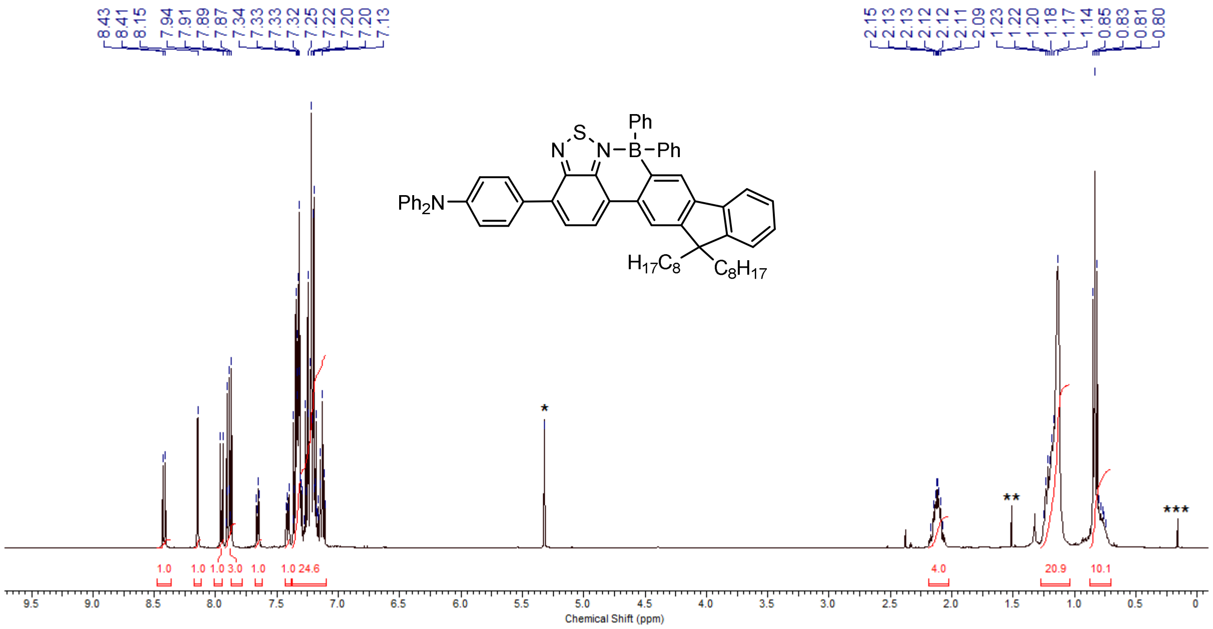

^{1}H NMR spectra of **4-BPh₂** in CD_2Cl_2. * = CH_2Cl_2, ** = H_2O, *** = Silicon Grease

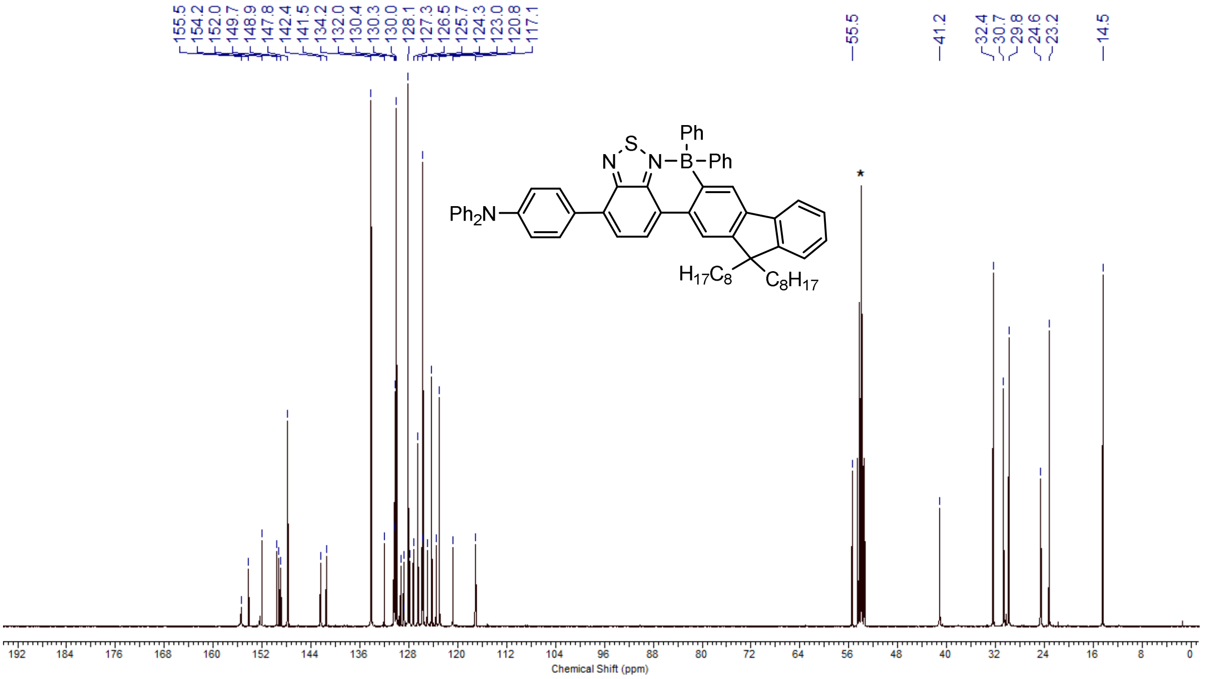

^{13}C{^{1}H} NMR spectra of **4-BPh₂** in CD_2Cl_2. * = CD_2Cl_2

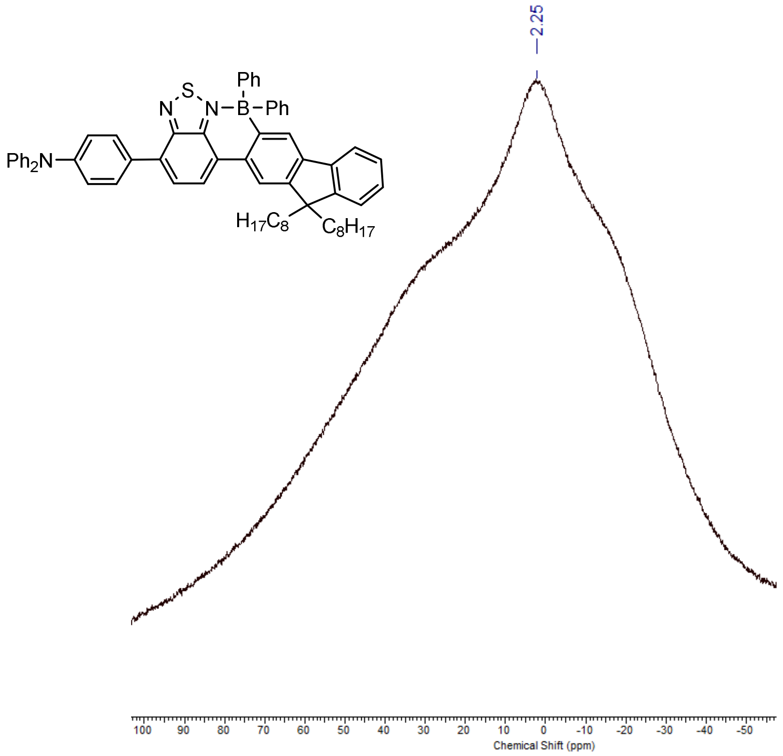

^{11}B NMR spectra of **4-BPh$_2$** in CD_2Cl_2

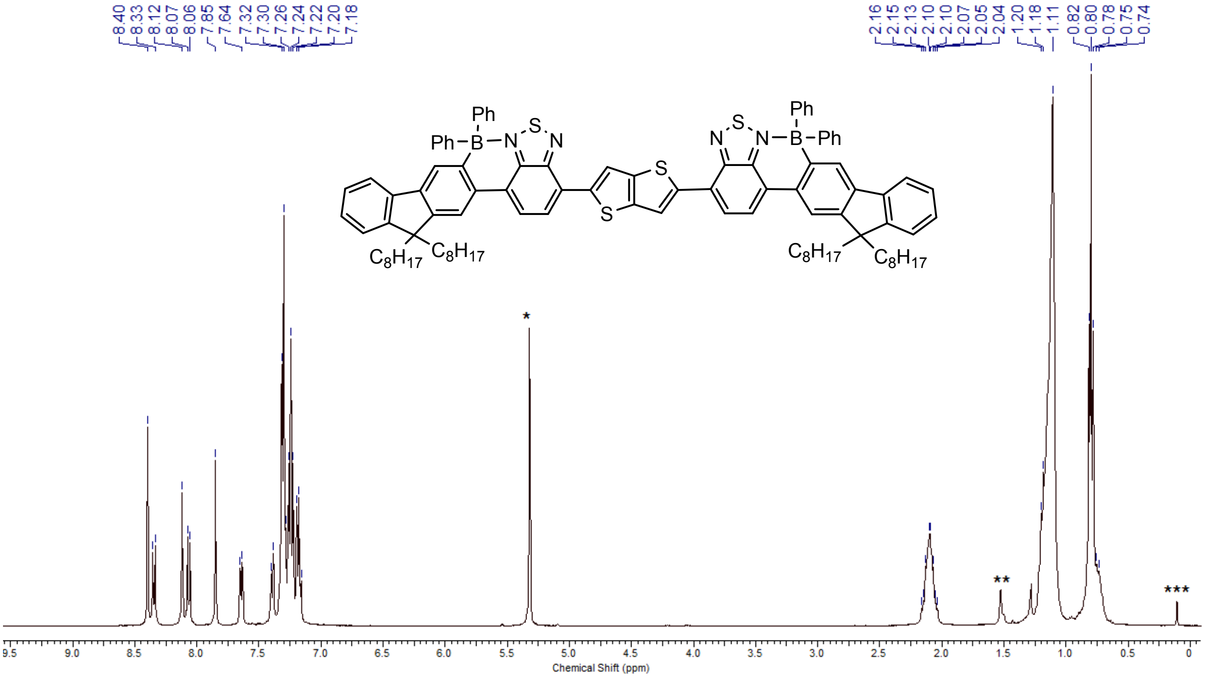

^{1}H NMR spectra of **5-BPh₂** in CD_2Cl_2. * = CH_2Cl_2, ** = H_2O, *** = Silicon Grease

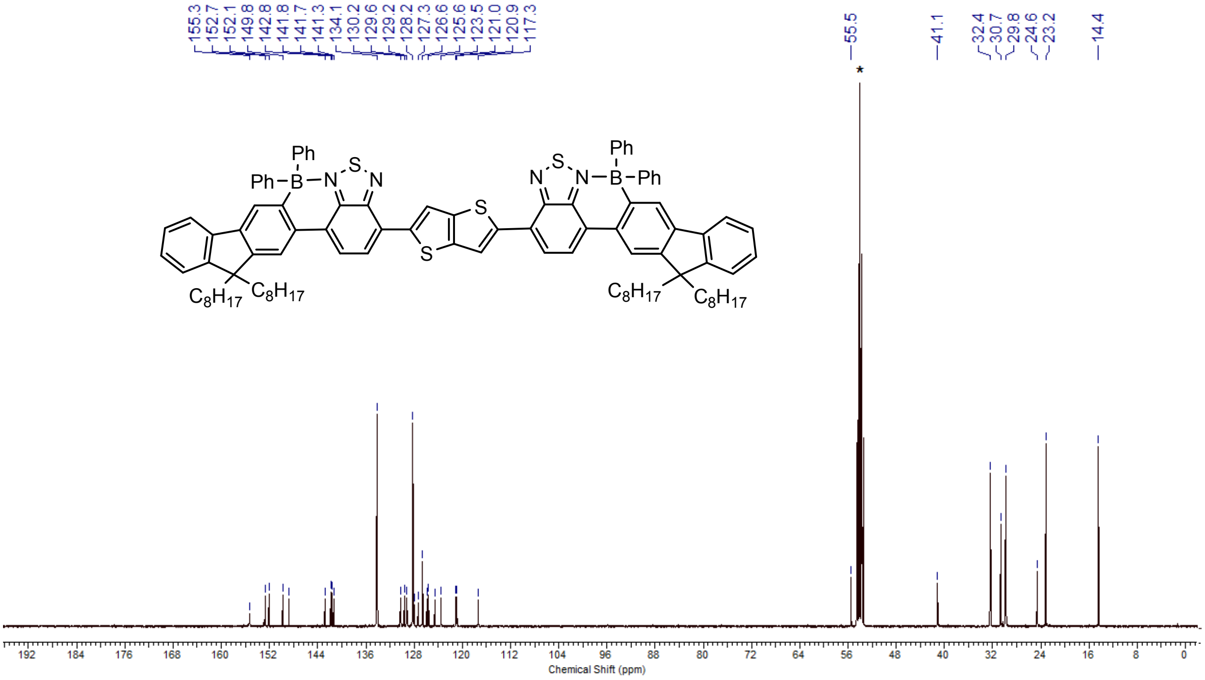

^{13}C{^{1}H} NMR spectra of **5-BPh₂** in CD_2Cl_2. * = CD_2Cl_2

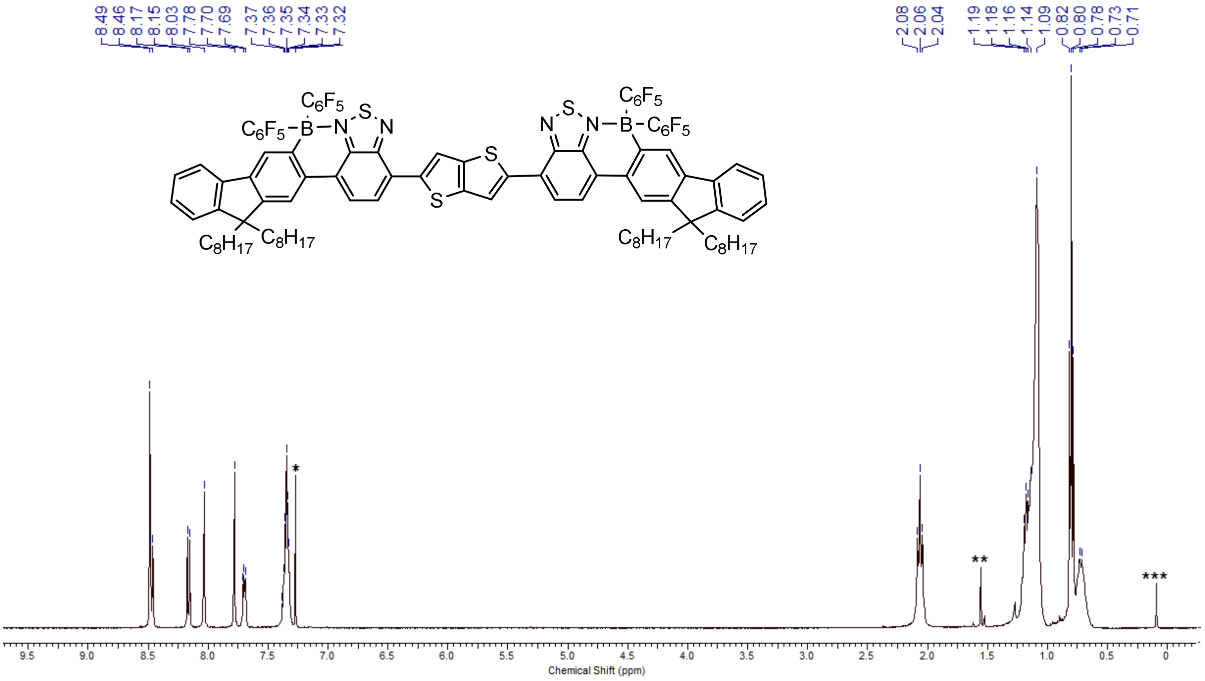

^{1}H NMR spectra of **5-B(C_6F_5)$_2$** in $CDCl_3$. * = $CHCl_3$, ** = H_2O, *** = Silicon Grease

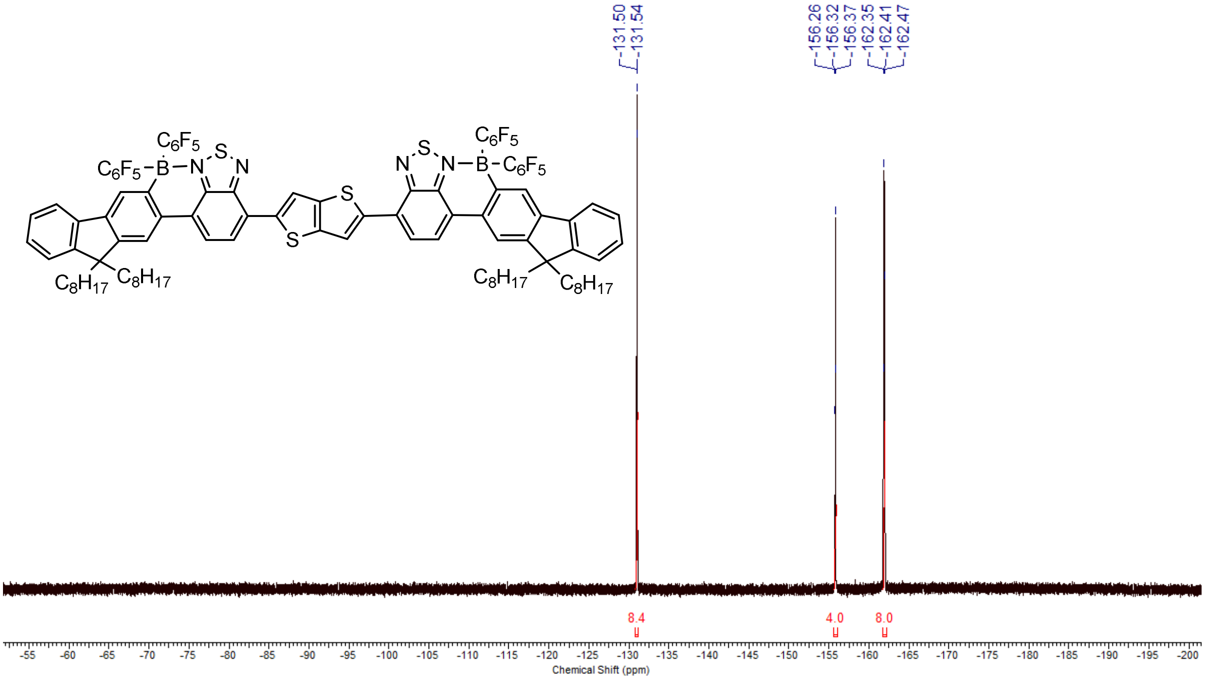

^{19}F NMR spectra of **5-B(C_6F_5)$_2$** in $CDCl_3$.

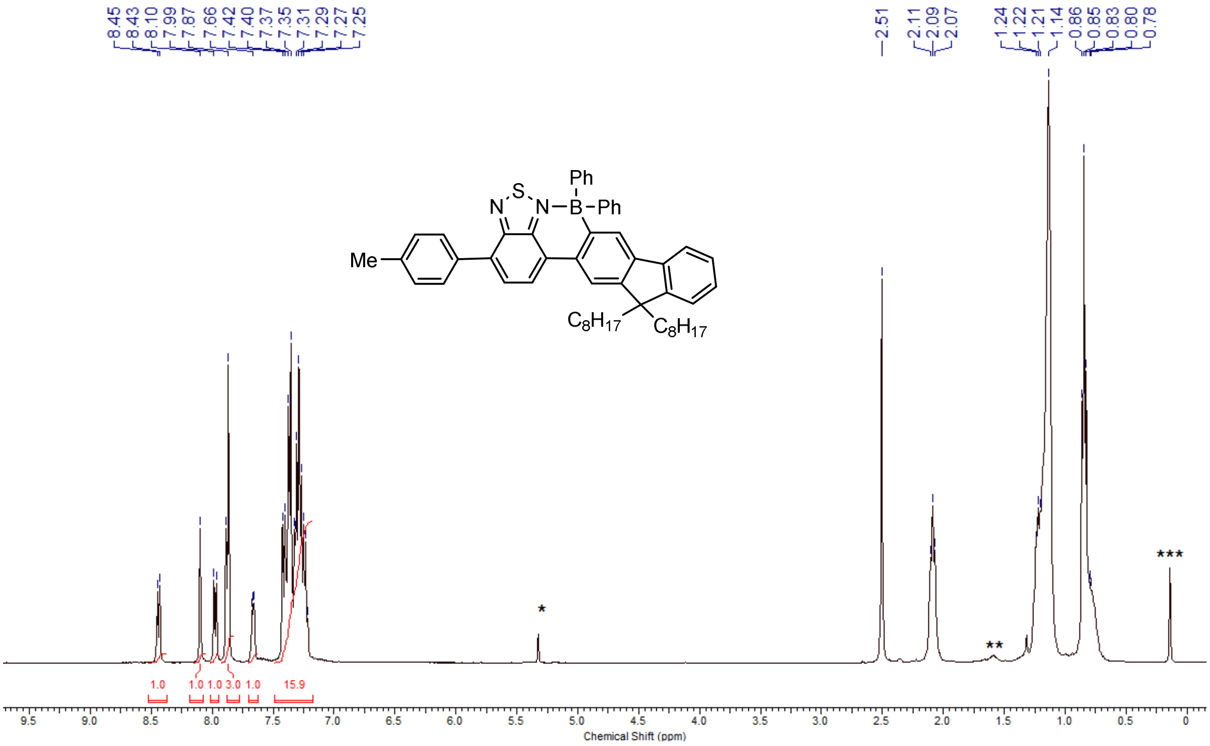

^1H NMR spectra of **6-BPh$_2$** in $CDCl_3$. * = $CHCl_3$, ** = H_2O, *** = Silicon Grease

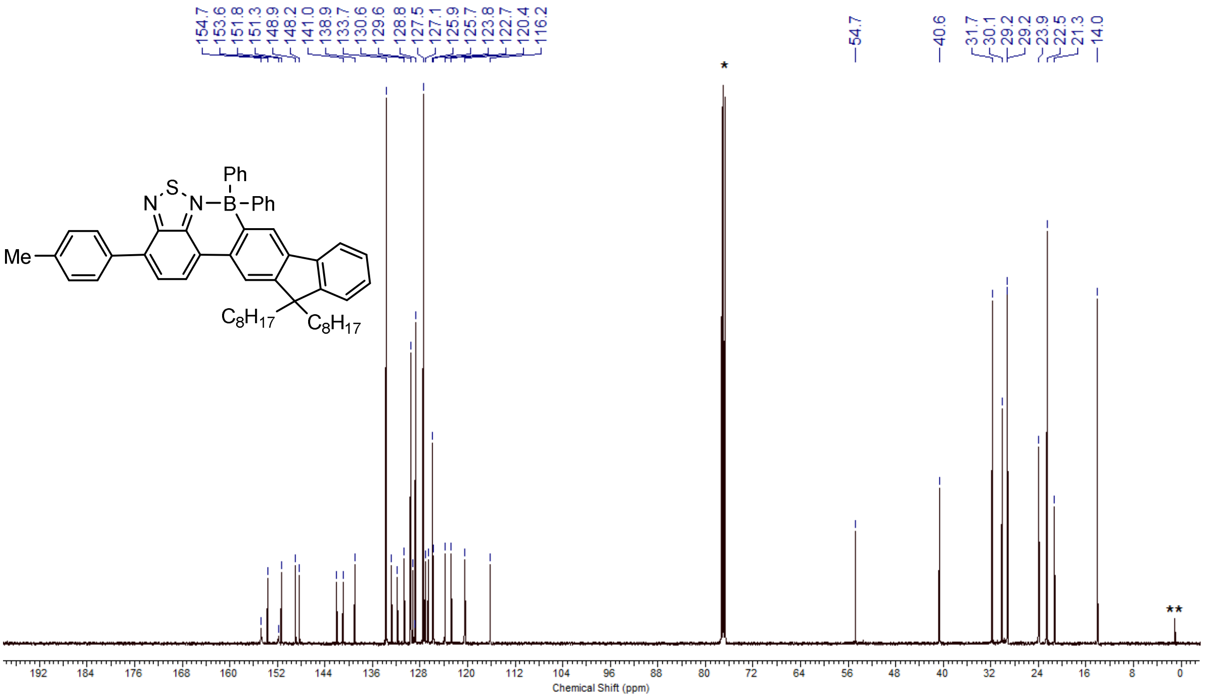

$^{13}C\{^1H\}$ NMR spectra of **6-BPh$_2$** in $CDCl_3$. * = $CDCl_3$, ** = Silicon Grease

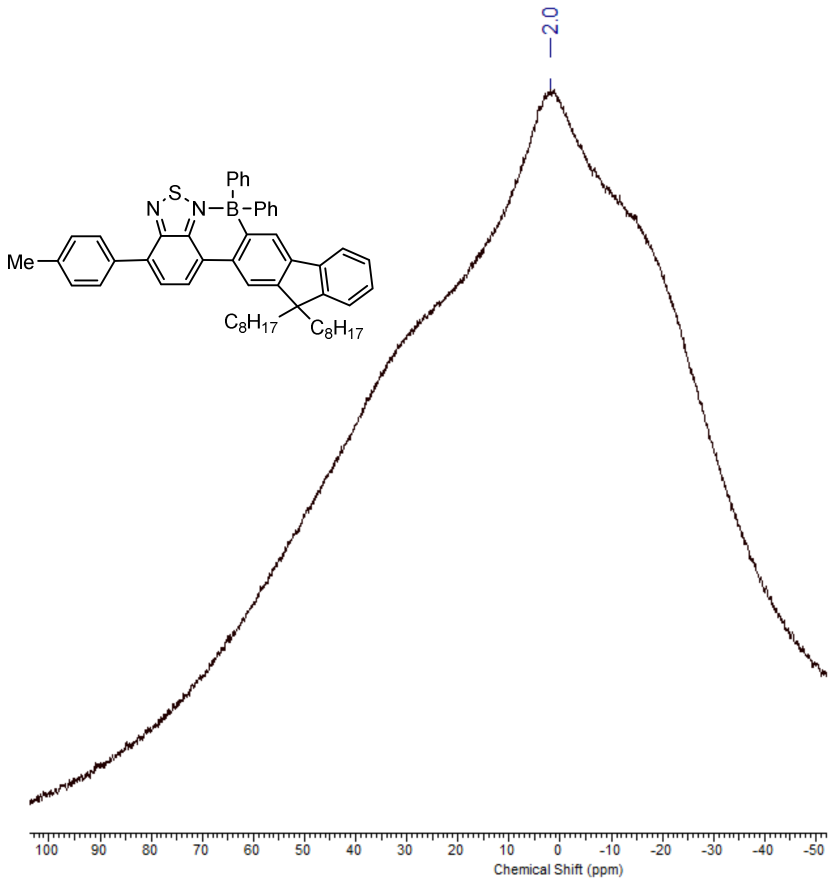

^{11}B NMR spectra of **6-BPh$_2$** in $CDCl_3$

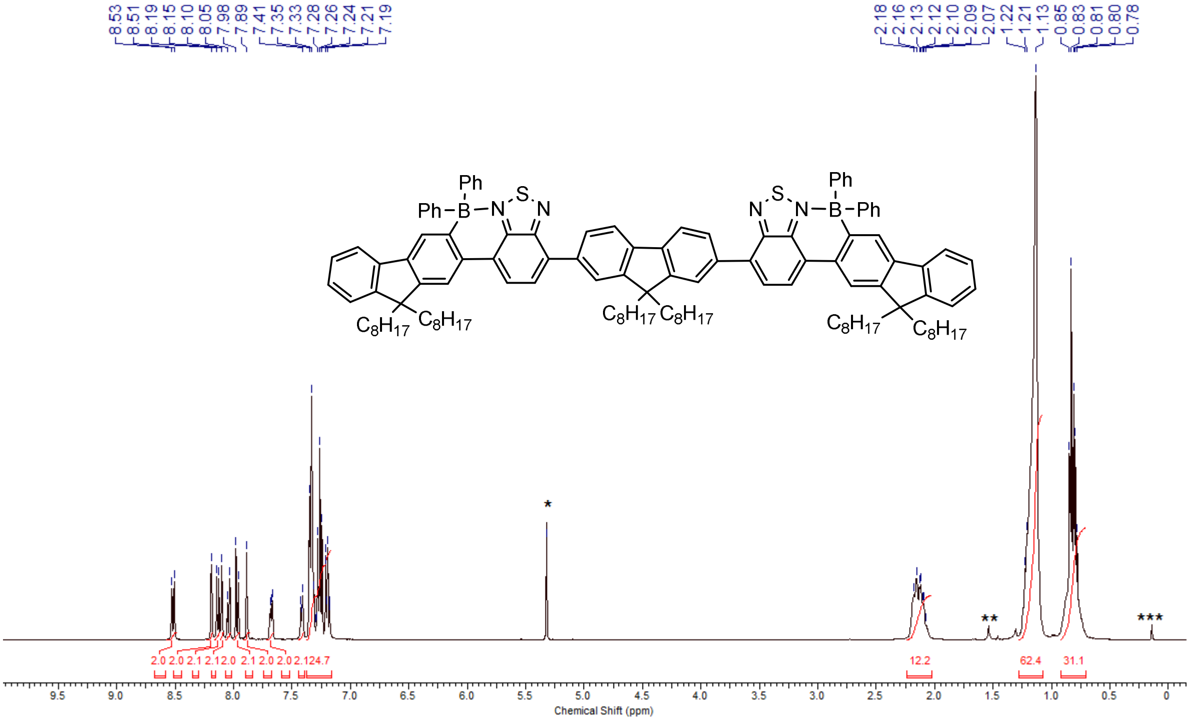

^{1}H NMR spectra of **7-BPh$_2$** in CD_2Cl_2. * = CH_2Cl_2, ** = H_2O, *** = Silicon Grease

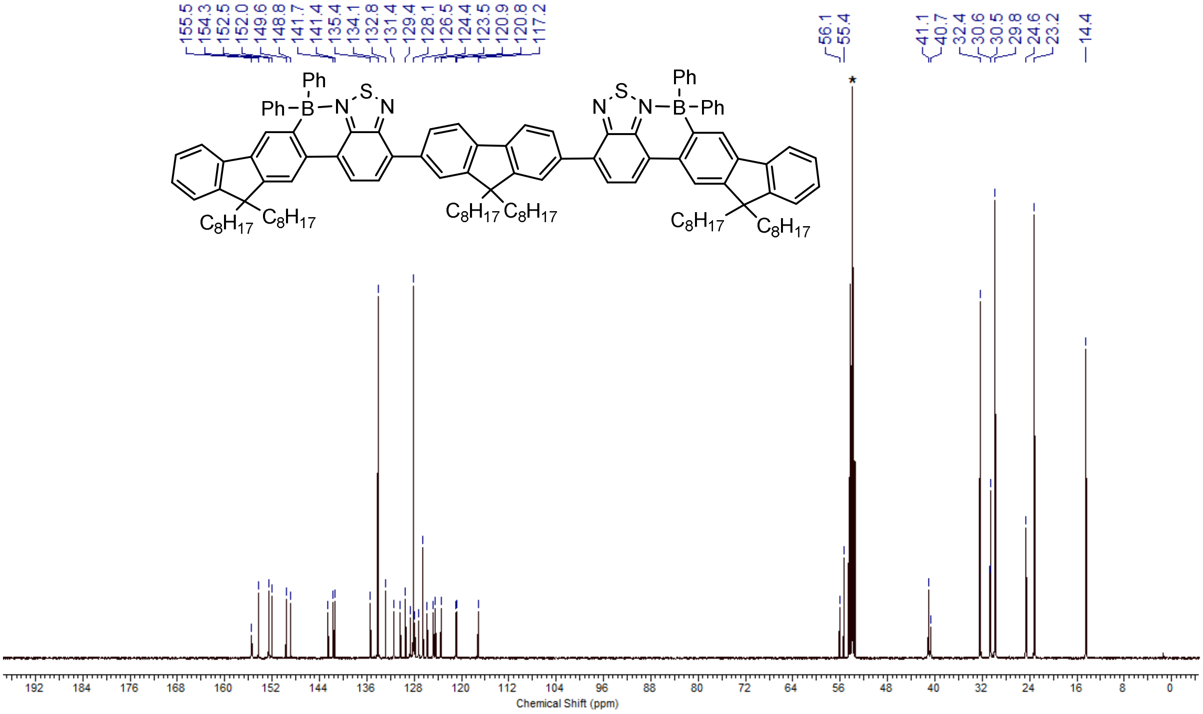

^{13}C{^{1}H} NMR spectra of **7-BPh$_2$** in CD_2Cl_2. * = CD_2Cl_2

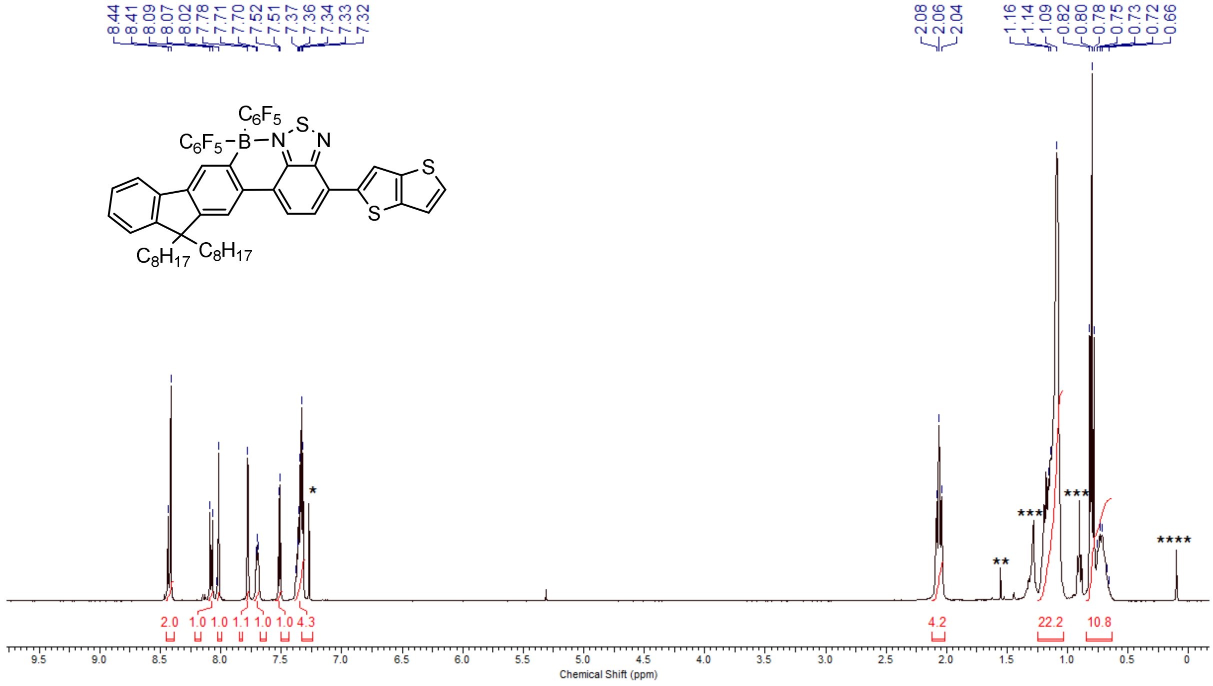

^{1}H NMR spectra of **8-B(C$_6$F$_5$)$_2$** in CDCl$_3$. * = CHCl$_3$, ** = H$_2$O, *** = Hexane, **** = Silicon Grease

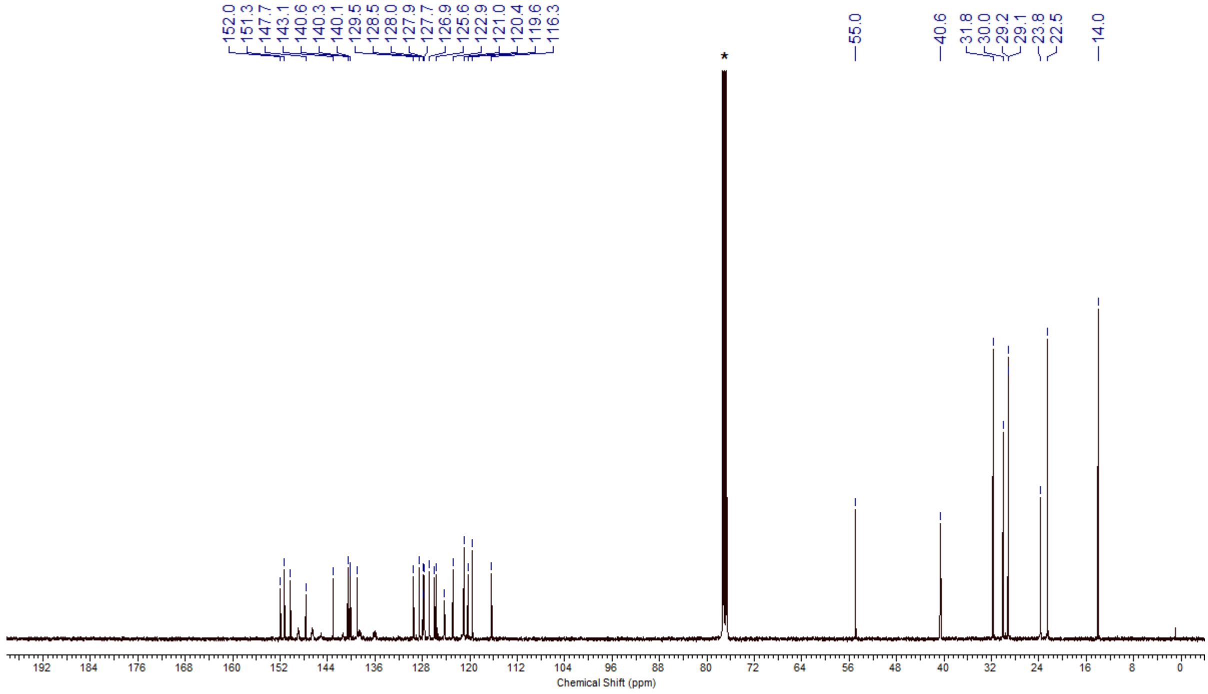

^{13}C{^{1}H} NMR spectra of **8-(C$_6$F$_5$)$_2$** in CDCl$_3$. * = CDCl$_3$

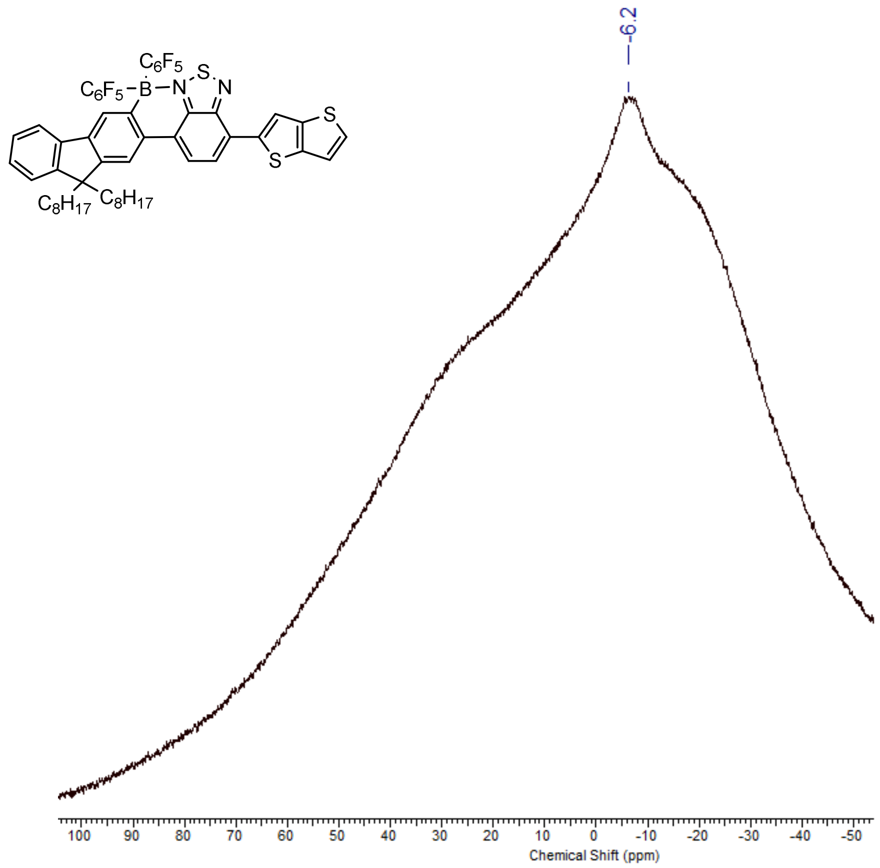

^{11}B NMR spectra of **8-B(C_6F_5)$_2$** in $CDCl_3$.

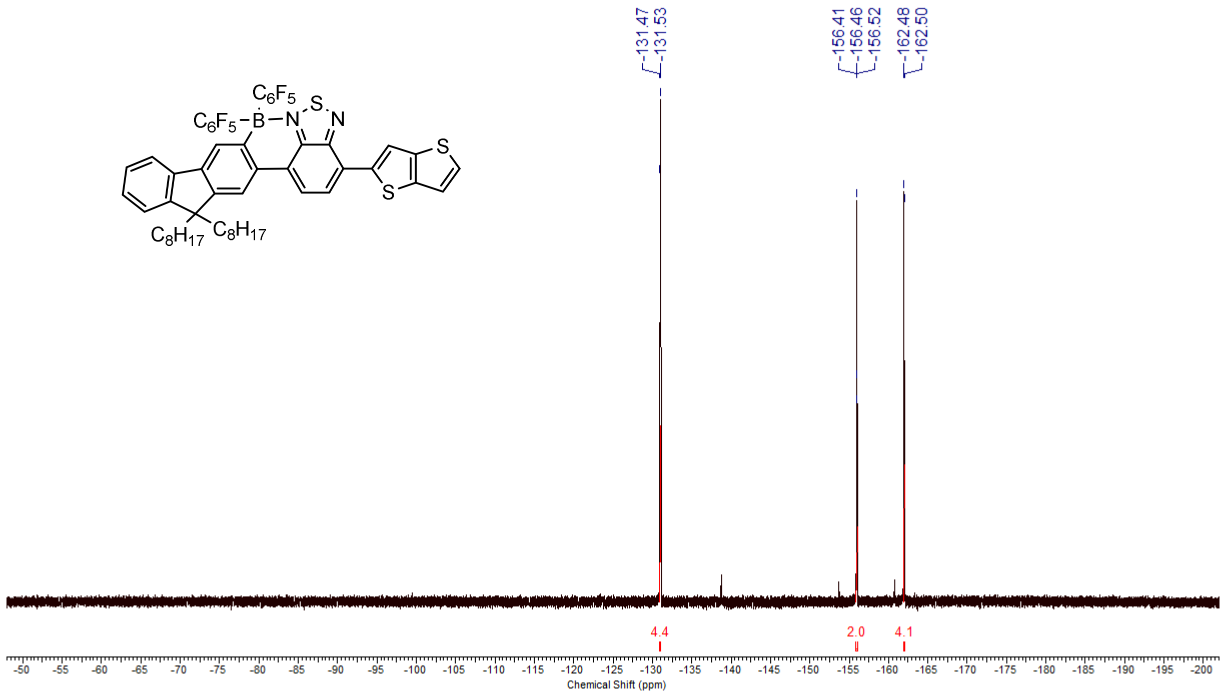

^{19}F NMR spectra of **8-B(C_6F_5)$_2$** in $CDCl_3$.

UV-vis and Fluorescence Spectra

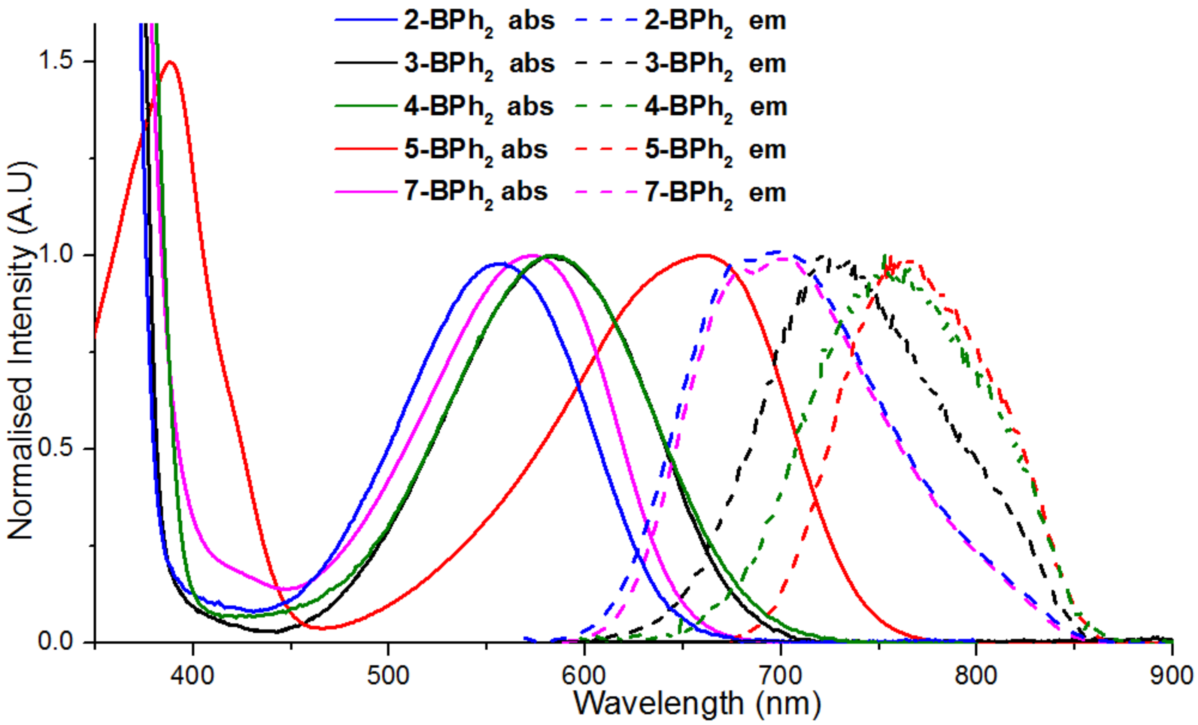

Figure S1: Uv-vis absorbance and fluorescence spectra of **2-BPh$_2$**, **3-BPh$_2$**, **4-BPh$_2$**, **5-BPh$_2$** and **7-BPh$_2$**.

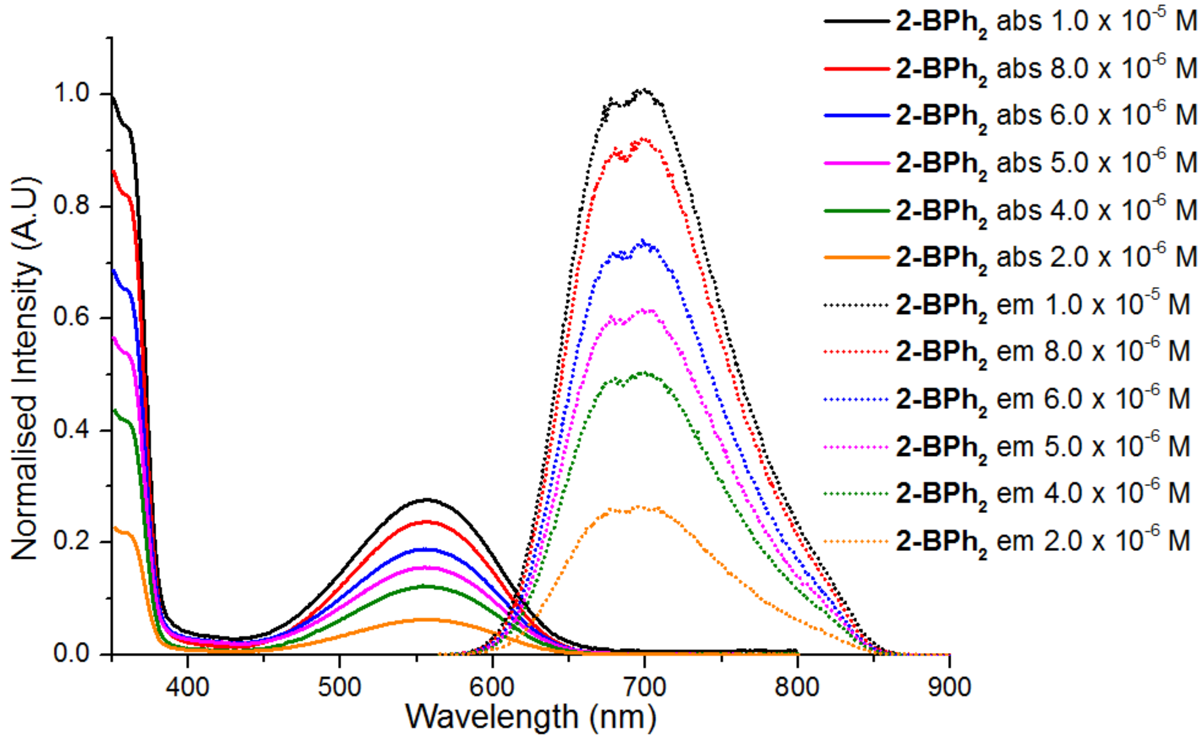

Figure S2: Absorbance and emission spectra of **2-BPh$_2$** at different concentrations.

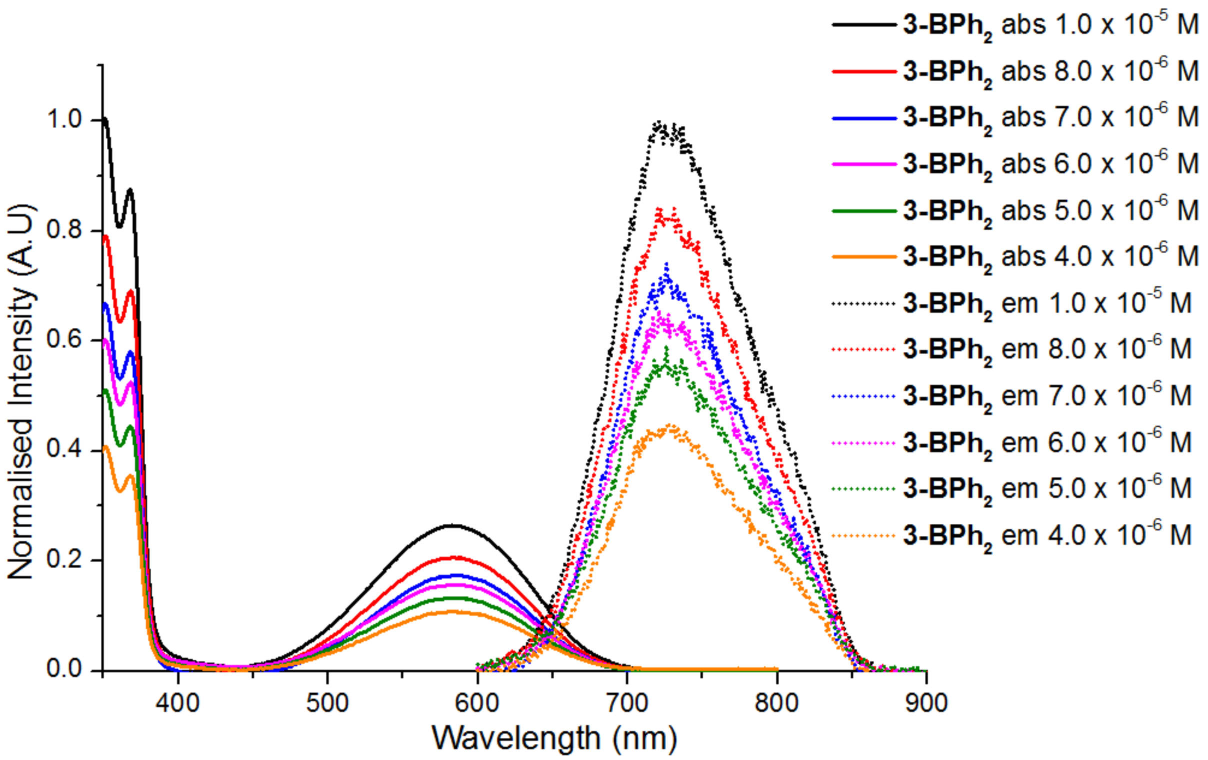

Figure S3: Absorbance and emission spectra of **3-BPh$_2$** at different concentrations.

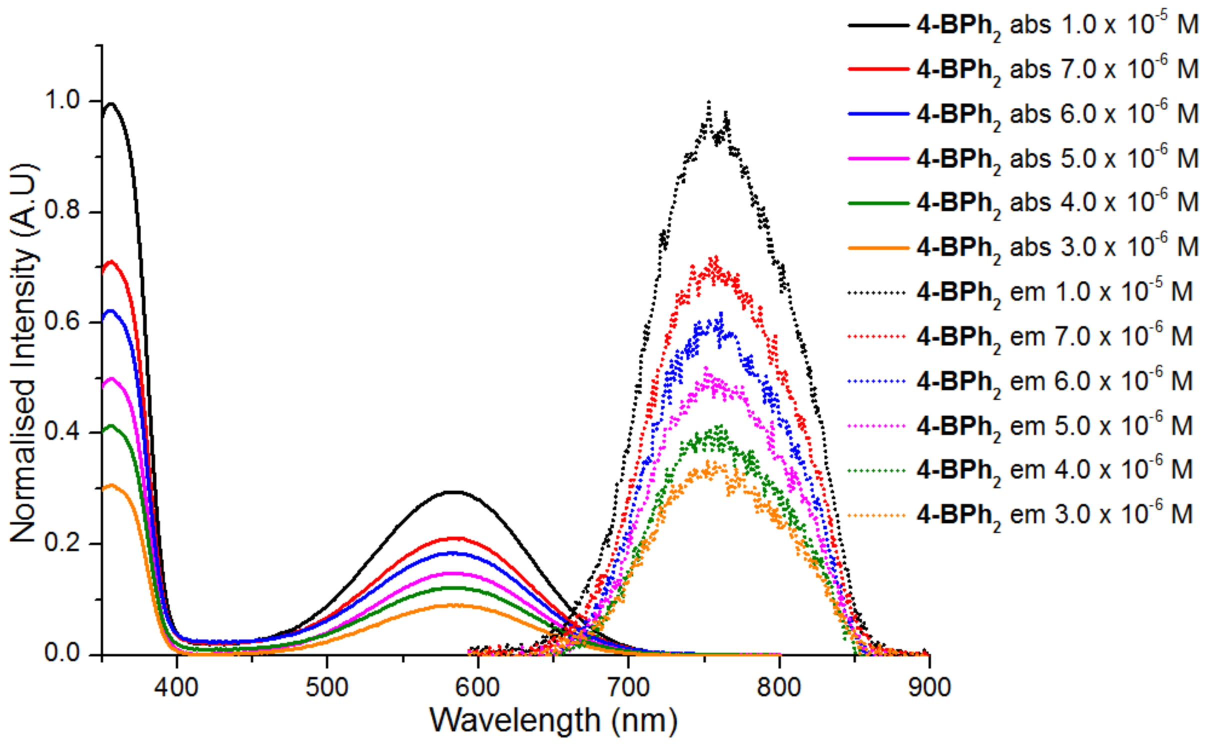

Figure S4: Absorbance and emission spectra of **4-BPh$_2$** at different concentrations.

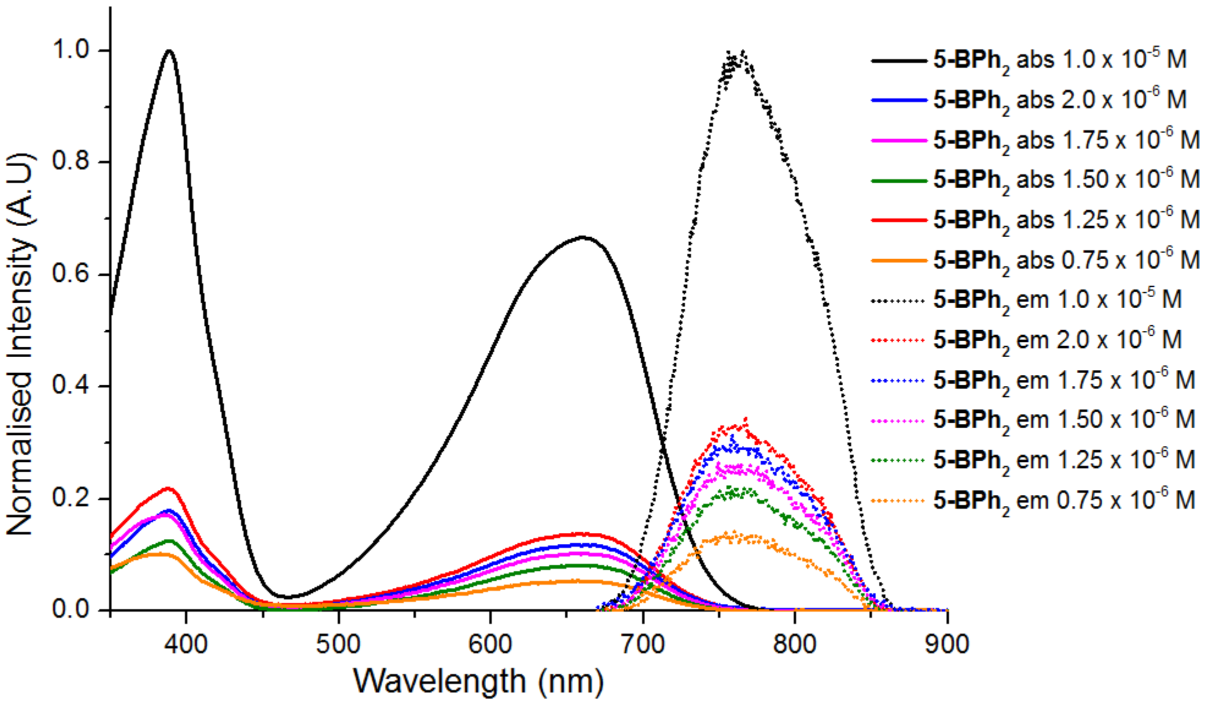

Figure S5: Absorbance and emission spectra of **5-BPh$_2$** at different concentrations.

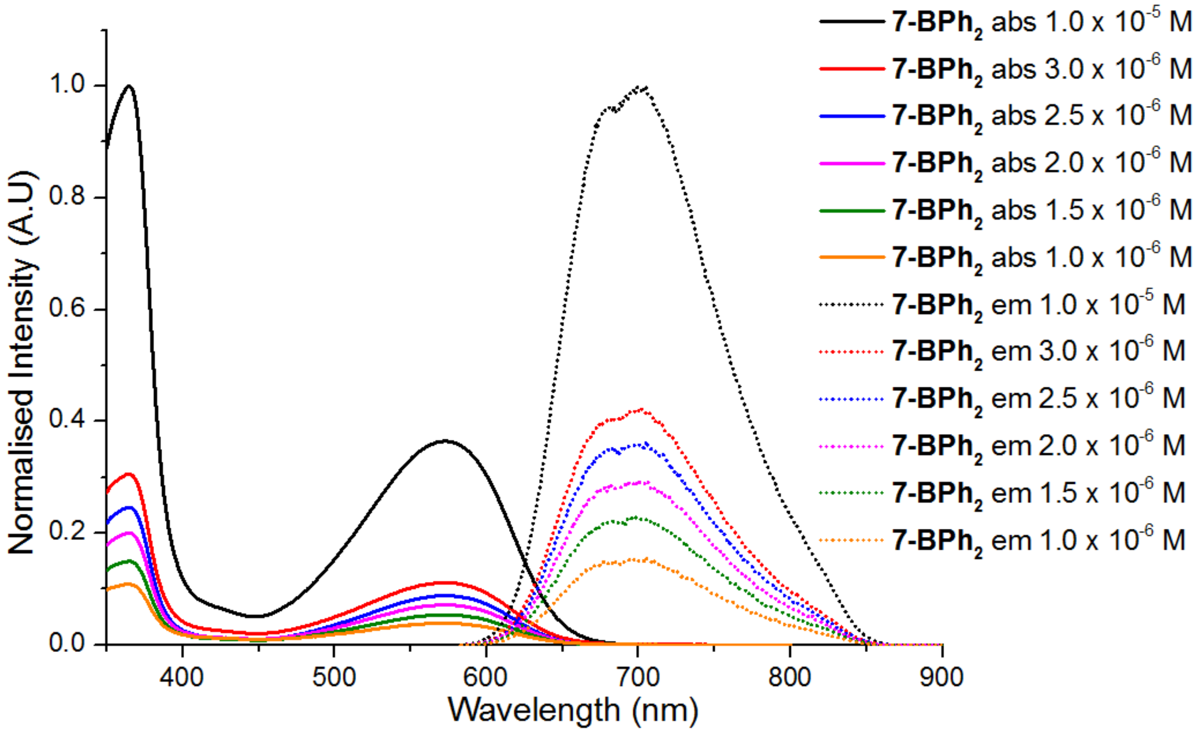

Figure S6: Absorbance and emission spectra of **7-BPh$_2$** at different concentrations.

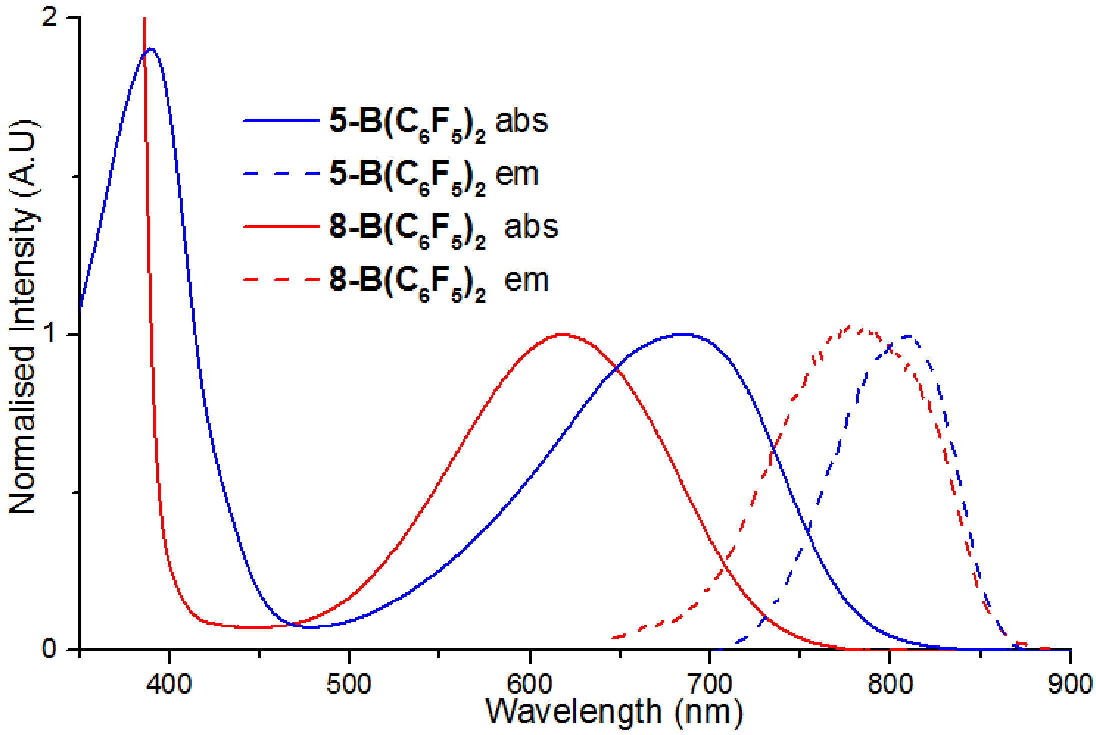

Figure S7: Uv-vis absorbance and fluorescence spectra of **5-B(C_6F_5)$_2$** and **8-B(C_6F_5)$_2$**.

Table S1: Comparison of photophysical properties of **5-B(C_6F_5)$_2$** and **8-B(C_6F_5)$_2$**

Compound	λmax$_{abs}$ (nm)	ε (M^{-1} cm^{-1})	Optical Band-Gap (eV)[a]	λmax$_{em}$ (nm)	Stokes shift (nm)
**5-B(C_6F_5)$_2$**	685	34300	1.59	805	120
**8-B(C_6F_5)$_2$**	619	15500	1.70	777	158

[a]Band-gap estimated from onset of absorption.

Cyclic Voltammograms

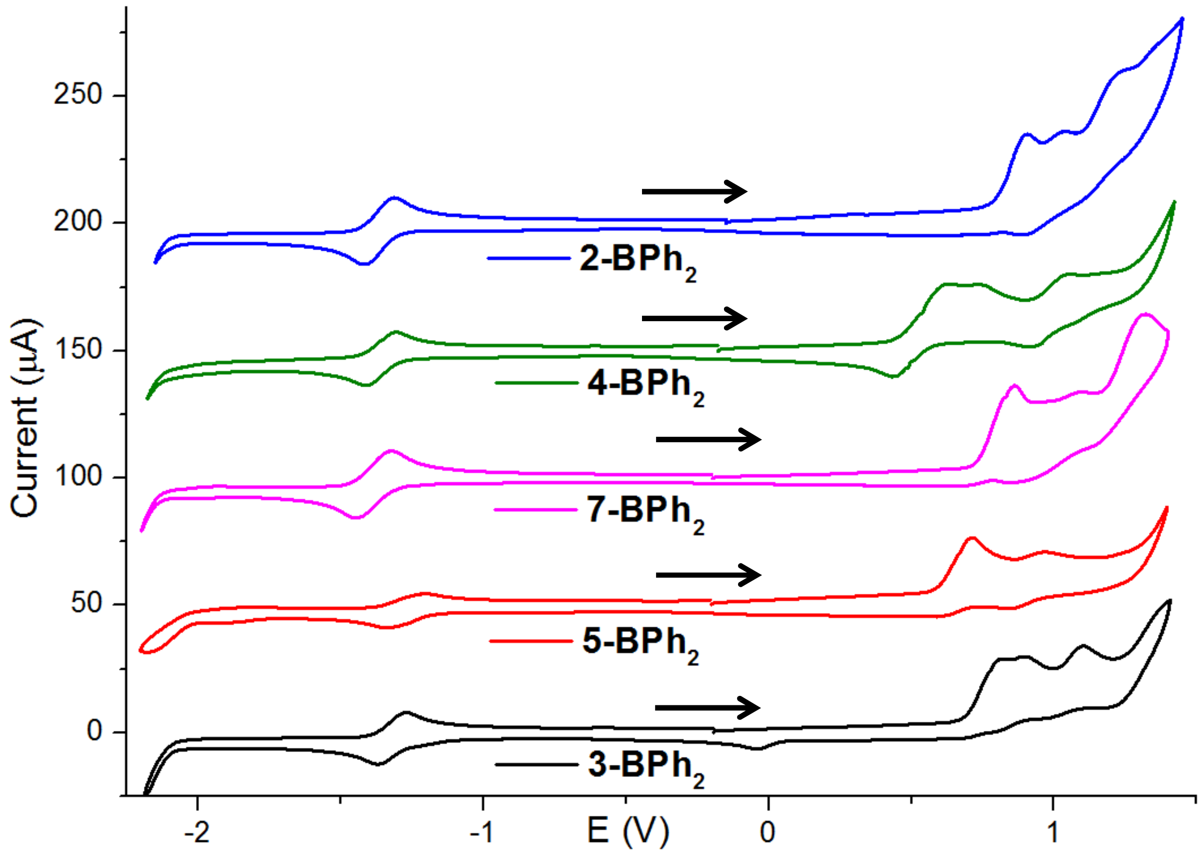

Figure S8: Cyclic voltammetry plots of **2-BPh$_2$**, **3-BPh$_2$**, **4-BPh$_2$**, **5-BPh$_2$** and **7-BPh$_2$**. (potential sweeps are offset by 50 μA for clarity). Measured in DCM, (1 mM), with [^{n}Bu$_4$N][PF$_6$] (0.1 M) as the supporting electrolyte at a scan rate of 50 mV s^{-1}.

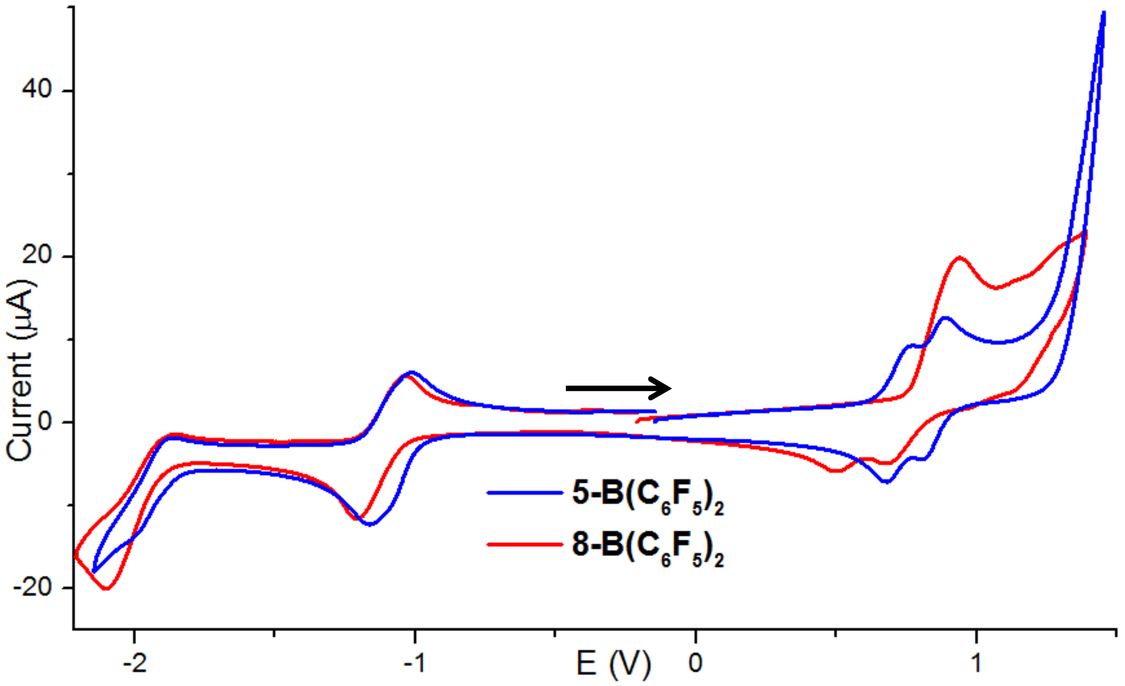

Figure S9: Cyclic voltammetry plots of **5-B(C_6F_5)$_2$** and **8-B(C_6F_5)$_2$**. Measured in DCM, (1 mM), with [^{n}Bu$_4$N][PF$_6$] (0.1 M) as the supporting electrolyte at a scan rate of 50 mV s^{-1}.

Table S2: Comparison of redox properties of **5-B(C_6F_5)$_2$** and **8-B(C_6F_5)$_2$**.

Compound	E_{ox}^{onset} (V)	E_{red}^{onset} (V)	HOMO (eV)	LUMO (eV)	Electrochemical Band-Gap (eV)
**5-B(C_6F_5)$_2$**	0.63	-0.95	-6.02	-4.44	1.58
**8-B(C_6F_5)$_2$**	0.76	-1.03	-6.15	-4.36	1.79

Measured in DCM (1 mM) with [^{n}Bu$_4$N][PF$_6$] (0.1 M) as the supporting electrolyte at a scan rate of 50 mV s^{-1}, potentials are given relative to Fc/Fc$^+$ redox couple which is taken to be 5.39 eV below vacuum.

Crystallographic Details

Table S3: Summary of crystallography data for **8-B(C_6F_5)$_2$**, **5-B(C_6F_5)$_2$** and **4-BPh$_2$**.

	**8-B(C_6F_5)$_2$**	**5-B(C_6F_5)$_2$**	**4-BPh$_2$**
**CCDC Reference**	1443404	1455079	1457709
**Empirical Formula**	$C_{53}H_{44}BF_{10}N_2S_3$	$C_{100}H_{86}B_2F_{20}N_4S_4$	$C_{65}H_{66}BN_3S$
**Fw / g mol^{-1}**	1005.93	1873.58	932.13
**Crystal System, Space group**	Orthorhombic, $P_{na}2_1$	Monoclinic, $P2_1$/n	Triclinic, P -1
***T* / K**	100	100	150
**a / Å**	26.2410(8)	17.3843(8)	10.6376(7)
**b / Å**	18.0986(7)	10.6799(4)	14.7722(1)
**c / Å**	9.9254(4)	24.0145(8)	18.1182(1)
**α / deg**	90.000	90.000	70.556(7)
**β / deg**	90.000	93.552(4)	74.499(6)
**γ / deg**	90.000	90.000	89.854(6)
**Vol / Å^3**	4713.8(3)	4450.2(3)	2575.4(4)
**Z**	4	2	2
**Calc. Density (Mg m^{-3})**	1.417	1.398	1.202
**Radiation / Å**	Cu *K*\α λ = 1.5418	λ = 0.6889	Mo *K*\α 0.71073
**Abs. Coeff. (mm^{-1})**	2.127	0.190	0.108
**F(000)**	2076	1936	996
**θ range (deg)**	5.9 - 130.1	3.3 - 49.0	6.6 - 52.7
**No. of data / Restraints / Parameters**	21658 / 7265	24167 / 8016	16176 / 10435
**R_{int}**	0.101	0.0685	0.0362
**No. of data / Restraints / Parameters**	7265 / 49 / 679	8016 / 119 / 636	10435 / 482 / 723
**R (data with [I^2 > 2σ(I^2)])**	5591 (0.0591)	4904 (0.1041)	10435 (0.0362)
**wR (all data)**	0.1291	0.2840	0.1706
**S**	0.975	1.088	1.020
**Δ*ρ*max, min / e·Å^{-3}**	0.54, -0.49	0.97, -0.54	0.66, -0.56

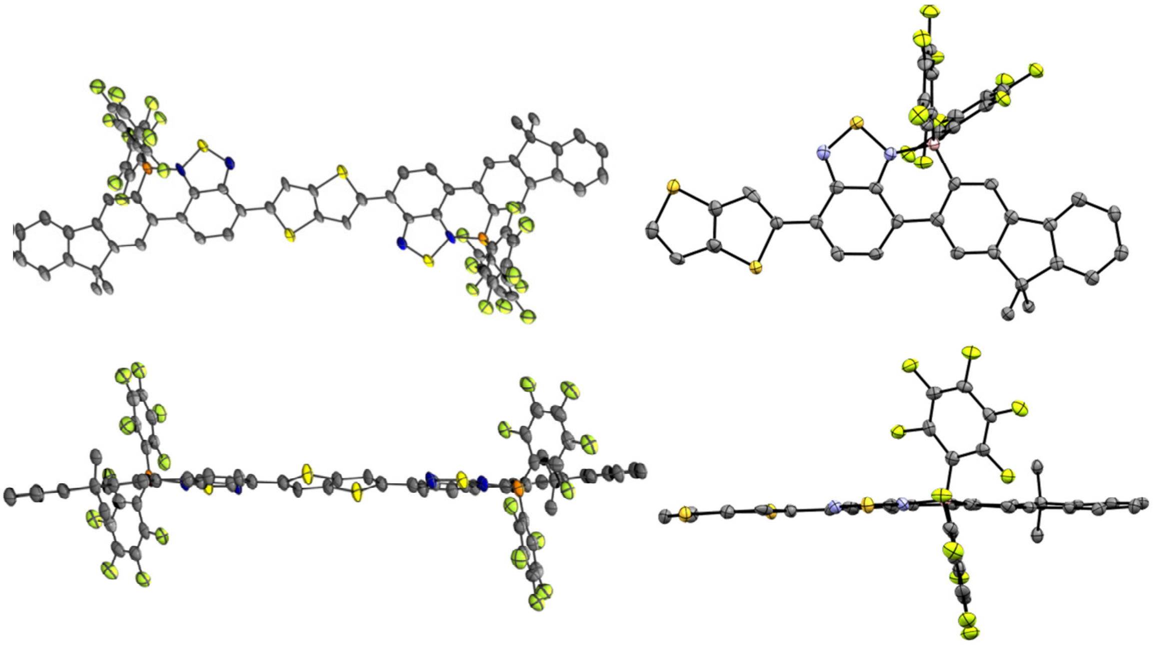

Figure S10: Solid state structures of **5-B(C_6F_5)$_2$** (left) and **8-B(C_6F_5)$_2$** (right), thermal ellipsoids at the 50 % probability level (hydrogens and octyl chains are omitted for clarity).

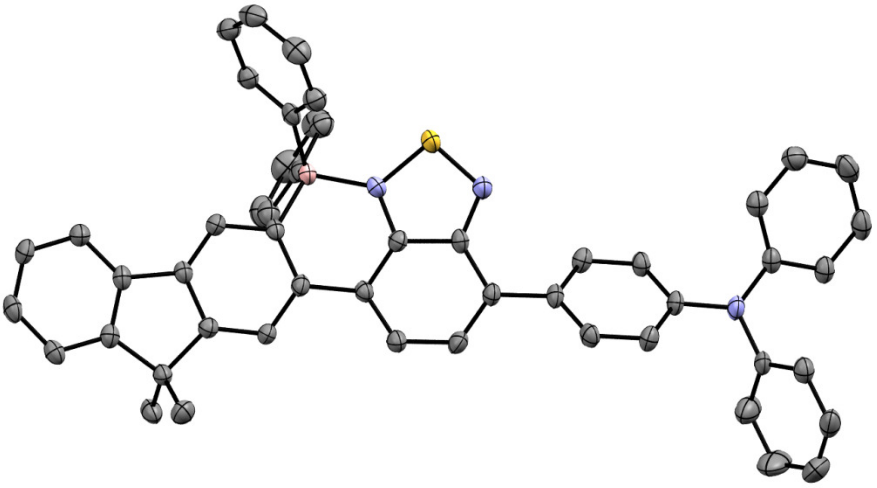

Figure S11: Solid state structure of **4-BPh$_2$**, thermal ellipsoids at the 50 % probability level (hydrogens and octyl chains are omitted for clarity).

Optimised Structure Coordinates

2’-BPh$_2$ Energy = -2383.68705263 A.U

	X	Y	Z
C	-7.379260000	3.879648000	-0.076489000
C	-7.335860000	1.107291000	0.418121000
C	-6.159897000	1.802417000	0.151824000
C	-6.180658000	3.182789000	-0.093460000
H	-7.404429000	4.947929000	-0.266172000
H	-7.320035000	0.040119000	0.609408000
C	-4.768302000	1.338515000	0.066488000
C	-3.954825000	2.443924000	-0.228838000
C	-4.211345000	0.078668000	0.229756000
C	-2.592471000	2.282720000	-0.354534000
C	-2.832292000	-0.119902000	0.089848000
H	-4.846010000	-0.771835000	0.464766000
C	-2.024847000	1.002742000	-0.203145000
H	-1.966162000	3.144101000	-0.561993000
C	-4.780065000	3.714994000	-0.355169000
C	-4.373835000	4.753261000	0.700562000
H	-3.343748000	5.079329000	0.535192000
H	-5.023402000	5.630216000	0.640453000
H	-4.448946000	4.333140000	1.705645000
C	-4.667971000	4.317603000	-1.763134000
H	-3.640088000	4.633054000	-1.959766000
H	-4.959372000	3.587947000	-2.521527000
H	-5.317436000	5.192186000	-1.853742000
C	-0.564329000	0.829753000	-0.316770000

C	0.005875000	-0.435469000	0.011181000
C	0.362023000	1.786927000	-0.651642000
C	1.418494000	-0.686788000	0.049063000
C	1.768279000	1.547485000	-0.654692000
H	0.034765000	2.780186000	-0.930942000
C	2.343085000	0.351374000	-0.307928000
H	2.412509000	2.363611000	-0.960873000
N	1.709733000	-1.922749000	0.447058000
S	0.353073000	-2.738042000	0.733000000
N	-0.687895000	-1.515958000	0.352037000
B	-2.291524000	-1.646789000	0.164197000
C	-2.474113000	-2.423946000	-1.257925000
C	-2.207126000	-3.797326000	-1.365505000
C	-2.840100000	-1.757392000	-2.432771000
C	-2.299790000	-4.473296000	-2.577525000
H	-1.935005000	-4.359827000	-0.475146000
C	-2.938480000	-2.423054000	-3.654016000
H	-3.056907000	-0.694129000	-2.397459000
C	-2.668056000	-3.784336000	-3.731044000
H	-2.090302000	-5.536281000	-2.623318000
H	-3.226013000	-1.876185000	-4.545318000
H	-2.744839000	-4.305688000	-4.678316000
C	-2.871053000	-2.447619000	1.446904000
C	-3.932320000	-3.355837000	1.341574000
C	-2.388056000	-2.184583000	2.737409000
C	-4.481145000	-3.975787000	2.462281000
H	-4.338223000	-3.585290000	0.361101000

C	-2.921100000	-2.803804000	3.863719000
H	-1.583352000	-1.465729000	2.870600000
C	-3.973208000	-3.705428000	3.728608000
H	-5.302789000	-4.673810000	2.345933000
H	-2.521559000	-2.579430000	4.846521000
H	-4.394164000	-4.189335000	4.602330000
C	-8.559582000	3.184522000	0.189581000
H	-9.503857000	3.715825000	0.206231000
C	-8.536878000	1.811036000	0.434632000
H	-9.463710000	1.288617000	0.639820000
C	3.804668000	0.139454000	-0.299544000
C	4.653893000	1.159326000	0.155341000
C	4.357416000	-1.060317000	-0.771363000
C	6.023559000	0.967958000	0.128595000
H	4.230548000	2.079414000	0.546853000
C	5.733144000	-1.251875000	-0.802838000
H	3.700460000	-1.842193000	-1.129898000
C	7.119763000	1.919093000	0.581572000
C	6.565091000	-0.233524000	-0.349249000
H	6.142851000	-2.182147000	-1.179910000
C	8.364536000	1.097369000	0.285428000
C	8.028783000	-0.152325000	-0.253276000
C	9.693099000	1.441257000	0.483129000
C	9.016200000	-1.070131000	-0.597928000
C	10.684942000	0.523185000	0.137426000
H	9.962950000	2.406590000	0.899225000
C	10.348707000	-0.720678000	-0.397579000

H	8.757707000	-2.037563000	-1.013940000
H	11.727805000	0.776918000	0.286332000
H	11.133462000	-1.420495000	-0.658932000
C	7.002233000	2.227551000	2.081547000
H	6.071607000	2.762085000	2.287755000
H	7.012319000	1.306652000	2.668000000
H	7.836316000	2.855487000	2.404285000
C	7.103661000	3.220561000	-0.232950000
H	7.179620000	3.010166000	-1.301682000
H	6.177454000	3.771424000	-0.050523000
H	7.943038000	3.857556000	0.056959000

3'-BPh$_2$ Energy = -2396.02310912 A.U

	**X**	**Y**	**Z**
C	6.342781000	-3.203474000	0.026538000
C	5.951019000	-0.442306000	0.420009000
C	4.868528000	-1.293067000	0.215644000
C	5.063901000	-2.668182000	0.020949000
H	6.502822000	-4.266376000	-0.123619000
H	5.801725000	0.620919000	0.572187000
C	3.426290000	-1.018330000	0.152566000
C	2.758309000	-2.231953000	-0.075980000
C	2.711769000	0.163701000	0.280688000
C	1.384359000	-2.255428000	-0.171774000
C	1.315668000	0.174343000	0.169576000
H	3.233618000	1.099419000	0.463238000
C	0.656977000	-1.055278000	-0.058127000

H	0.873425000	-3.199713000	-0.326325000
C	3.739316000	-3.388715000	-0.177737000
C	3.501023000	-4.427398000	0.928066000
H	2.517616000	-4.889692000	0.809537000
H	4.256864000	-5.215233000	0.876119000
H	3.551001000	-3.961452000	1.914300000
C	3.670151000	-4.057151000	-1.558637000
H	2.689721000	-4.516814000	-1.708545000
H	3.835427000	-3.324640000	-2.351422000
H	4.429759000	-4.838565000	-1.642555000
C	-0.814379000	-1.080003000	-0.147241000
C	-1.542519000	0.110687000	0.142242000
C	-1.612426000	-2.162559000	-0.428486000
C	-2.976139000	0.178158000	0.200350000
C	-3.034843000	-2.110117000	-0.417788000
H	-1.162216000	-3.115846000	-0.673473000
C	-3.762319000	-0.987408000	-0.101526000
H	-3.563845000	-3.019803000	-0.677454000
N	-3.419082000	1.383527000	0.543032000
S	-2.176419000	2.381115000	0.766380000
N	-0.991421000	1.286507000	0.420970000
B	0.579714000	1.618583000	0.201522000
C	0.641121000	2.364290000	-1.247483000
C	0.203122000	3.689298000	-1.394857000
C	1.070564000	1.708095000	-2.406714000
C	0.192116000	4.328349000	-2.630112000
H	-0.122217000	4.245299000	-0.518439000

C	1.066243000	2.337322000	-3.650727000
H	1.421295000	0.682813000	-2.340137000
C	0.625908000	3.650720000	-3.767145000
H	-0.149847000	5.354532000	-2.707156000
H	1.406438000	1.799915000	-4.529154000
H	0.621739000	4.144247000	-4.732194000
C	1.060878000	2.530809000	1.450497000
C	1.990850000	3.568236000	1.304958000
C	0.623405000	2.250112000	2.753465000
C	2.456583000	4.294337000	2.399194000
H	2.357264000	3.817009000	0.313698000
C	1.074351000	2.972877000	3.853487000
H	-0.080723000	1.437918000	2.916905000
C	1.995002000	4.002467000	3.678456000
H	3.177917000	5.090589000	2.252087000
H	0.712801000	2.731102000	4.846756000
H	2.349827000	4.569223000	4.531579000
C	7.428970000	-2.351858000	0.229613000
H	8.434738000	-2.755016000	0.236978000
C	7.233501000	-0.984329000	0.425294000
H	8.088951000	-0.338269000	0.583199000
C	-5.220268000	-0.958908000	-0.071432000
C	-6.065848000	0.115648000	-0.161225000
S	-6.133413000	-2.438675000	0.060348000
C	-7.439529000	-0.251007000	-0.133425000
H	-5.713494000	1.131888000	-0.254828000
C	-7.643547000	-1.595068000	-0.018273000

H	-8.254806000	0.457650000	-0.198934000
C	-8.942757000	-2.334130000	0.053523000
H	-9.044090000	-2.868849000	1.000304000
H	-9.766326000	-1.624843000	-0.029658000
H	-9.032733000	-3.062789000	-0.754630000

4'-BPh$_2$ Energy = -2553.31364707 A.U

	**X**	**Y**	**Z**
C	-9.412680000	1.384689000	-0.574488000
C	-9.513328000	2.776120000	-0.588109000
C	-8.372599000	3.573280000	-0.486270000
C	-7.134478000	2.959590000	-0.371754000
C	-7.035097000	1.560999000	-0.359319000
C	-8.171616000	0.764167000	-0.460479000
H	-10.310096000	0.782414000	-0.652769000
H	-10.487927000	3.241216000	-0.679479000
H	-8.459590000	4.655083000	-0.497841000
H	-8.095292000	-0.317427000	-0.450678000
C	-5.764108000	3.607946000	-0.245955000
C	-4.866183000	2.383431000	-0.162884000
C	-3.493651000	2.323489000	-0.054717000
C	-2.853695000	1.070943000	0.002125000
C	-3.596952000	-0.129578000	-0.066054000
C	-4.988471000	-0.036919000	-0.189893000
C	-5.616733000	1.199233000	-0.231021000
H	-2.915601000	3.241375000	-0.029919000
H	-5.575371000	-0.949578000	-0.251628000

C	-5.435584000	4.460954000	-1.479707000
H	-6.138831000	5.293491000	-1.563522000
H	-4.426657000	4.873478000	-1.397788000
H	-5.493844000	3.862962000	-2.391498000
C	-5.669084000	4.458906000	1.029002000
H	-4.660050000	4.863346000	1.141961000
H	-6.369002000	5.296797000	0.978521000
H	-5.905806000	3.861298000	1.911717000
C	-1.382817000	1.003818000	0.085835000
C	-0.741426000	-0.256983000	-0.086479000
C	-0.511367000	2.053185000	0.247091000
C	0.682389000	-0.423417000	-0.169660000
C	0.905357000	1.903132000	0.214519000
H	-0.894934000	3.052760000	0.406473000
C	1.549252000	0.711001000	-0.009153000
H	1.501937000	2.791614000	0.384698000
C	3.018312000	0.607055000	-0.076193000
C	3.772062000	1.661642000	-0.606924000
C	3.706363000	-0.509268000	0.414862000
C	5.154812000	1.613328000	-0.637183000
H	3.267166000	2.520429000	-1.034241000
C	5.091197000	-0.560155000	0.396744000
H	3.156615000	-1.335002000	0.846817000
C	5.835324000	0.501845000	-0.127106000
H	5.718175000	2.433435000	-1.065958000
H	5.605403000	-1.422944000	0.802768000
N	-1.376516000	-1.416054000	-0.223312000

N	1.036393000	-1.685267000	-0.401204000
S	-0.273044000	-2.615638000	-0.483721000
N	7.244234000	0.462236000	-0.140095000
C	7.983497000	1.635910000	0.157358000
C	9.130081000	1.953788000	-0.575236000
C	7.573564000	2.481548000	1.191284000
C	9.856167000	3.098273000	-0.269355000
H	9.447840000	1.299740000	-1.378807000
C	8.295904000	3.632804000	1.478611000
H	6.688845000	2.231444000	1.765237000
C	9.442506000	3.945843000	0.754343000
H	10.744132000	3.334156000	-0.843923000
H	7.967206000	4.279999000	2.283073000
H	10.007825000	4.840070000	0.985925000
C	7.924782000	-0.753112000	-0.401367000
C	9.066844000	-1.095075000	0.328190000
C	7.464420000	-1.618098000	-1.398135000
C	9.739583000	-2.279506000	0.054877000
H	9.424279000	-0.428156000	1.103852000
C	8.133705000	-2.808510000	-1.651964000
H	6.583088000	-1.351983000	-1.970120000
C	9.276839000	-3.144584000	-0.932193000
H	10.624614000	-2.531825000	0.627061000
H	7.765649000	-3.470024000	-2.427243000
H	9.800578000	-4.069682000	-1.138428000
B	-2.953363000	-1.608525000	0.100964000
C	-2.995191000	-2.116591000	1.650538000

C	-2.582654000	-3.414073000	1.989861000
C	-3.392781000	-1.284545000	2.703390000
C	-2.562077000	-3.860860000	3.306768000
H	-2.287824000	-4.104493000	1.202849000
C	-3.380720000	-1.721008000	4.028008000
H	-3.723148000	-0.272844000	2.489028000
C	-2.962492000	-3.010216000	4.334909000
H	-2.240466000	-4.871611000	3.532402000
H	-3.697462000	-1.051373000	4.820043000
H	-2.951736000	-3.353031000	5.363217000
C	-3.546671000	-2.672093000	-0.965865000
C	-4.516157000	-3.618754000	-0.611750000
C	-3.176368000	-2.619467000	-2.318028000
C	-5.086521000	-4.472504000	-1.553799000
H	-4.832799000	-3.691277000	0.424112000
C	-3.730802000	-3.473337000	-3.266919000
H	-2.445966000	-1.883037000	-2.644376000
C	-4.691065000	-4.406293000	-2.885666000
H	-5.836976000	-5.192291000	-1.245944000
H	-3.419384000	-3.407446000	-4.303531000
H	-5.127324000	-5.073436000	-3.620227000
